# The beneficial impact of silicon on wheat drought resilience is dependent on cultivar and stress intensity

**DOI:** 10.3389/fpls.2025.1661405

**Published:** 2025-08-21

**Authors:** Katie Shaw, Sarah Thorne, Caroline Chapman, Andrew Fleming, Susan Hartley, Julie Gray

**Affiliations:** ^1^ School of Biosciences, University of Sheffield, Sheffield, United Kingdom; ^2^ Department of Biology, University of York, York, United Kingdom

**Keywords:** drought, gas exchange, genotype, silicon, stomatal density, water use, wheat

## Abstract

Drought has a major impact on crop yields. Silicon (Si) application has been proposed to improve drought resilience via several mechanisms including modifying the level of stomatal gas exchange. However, the impact of Si on transpiration and stomatal conductance varies between studies. We assessed the impact of supplemental Si on wheat water use and drought resilience in two high Si accumulating genotypes that vary in stomatal density and stomatal conductance. These genotypes varied considerably in their responses to Si treatment and short-term severe drought at the booting stage of development. For example, gas exchange measurements revealed that one genotype (H5) showed a significant increase in stomatal conductance with Si treatment, but the other genotype (H3) did not. Application of Si increased yield 3.5-fold in the H5 higher stomatal density genotype following the severe drought but Si had no yield-effect on the H3 lower stomatal density genotype. To determine whether differences in stomatal density could account for these differing Si responses, a modern cultivar, Fielder, was grown alongside a reduced stomatal density mutant, *TaEPF1OE*. Gas exchange measurements again showed that Si had no impact on the stomatal conductance of the lower stomatal density genotype, *TaEPF1OE*, but did increase stomatal conductance in the Fielder background. This is in line with the results from H3 and H5, suggesting that stomatal density plays an important role in the impact of Si treatment on stomatal function. However, following severe drought, Si increased yields in both the *TaEPF1OE* stomatal density mutant and the Fielder background, indicating that stomatal density alone does not account for genotype-specific yield responses seen in H3 and H5. Next, two genotypes that showed yield improvements with Si under short-term severe drought stress (Fielder and H5) were subjected to a longer-term vegetative drought stress. Here, Si had minimal effects on stomatal conductance, water use or biomass, suggesting that the impact of Si on drought resilience is strongly affected by drought type and duration. We conclude that for Si fertilization to be used as an effective drought mitigation strategy, crop cultivar, together with drought intensity and duration, must be considered.

## 1 Introduction

Producing sufficient food to sustainably feed an estimated population of 9.7 billion people by 2050 is a significant challenge, particularly as yield trends are insufficient to meet projected global demands and the impacts of climate change on agriculture are increasing (Ray et al., 2013; Challinor et al., 2014; Cole et al., 2018). The increased frequency and severity of extreme weather events, such as drought, present a major threat to agriculture due to its sensitivity to weather parameters (Malhi et al., 2021; IPCC, 2023). For example, from 1964 to 2007, drought periods reduced global cereal yields by 5.1% and harvested area by 4.1% (Lesk et al., 2016). Wheat plays an essential role in global food security, providing the global population with approximately one fifth of dietary calories and proteins (Erenstein et al., 2022). However, its yields are susceptible to drought (Zampieri et al., 2017). Drought stress can reduce wheat yields at all stages of development, although terminal drought, where drought occurs during the flowering and grain-filling stages of development, typically causes the largest reduction in yields (Farooq et al., 2014). The intensity and duration of drought are also important contributors to the impact of drought stress on wheat yields (Farooq et al., 2014; Zhang et al., 2018). Consequently, there is an urgent need to improve the drought resilience of wheat.

Silicon (Si) fertilization has been shown to provide improved resilience against both drought and other stressors including salinity, high ultraviolet (UV) radiation, heavy metal toxicity, nutrient imbalances, pathogens and herbivory (reviewed by e.g. Coskun et al., 2016; Debona et al., 2017; Luyckx et al., 2017; Frew et al., 2018; Thorne et al., 2020). A range of mechanisms have been proposed for the alleviation of drought stress by Si; these include modifying gas exchange, reducing oxidative damage, improving photosynthetic rate, improving water uptake from the soil, increasing mineral nutrient uptake and regulating phytohormone synthesis. Whilst Si is the second most abundant element in soils, it is only accessible to plants as monosilicic acid, Si(OH)_4_ (Raven, 1983; Gocke et al., 2013). This form of Si is soluble in the soil at pH< 9 and concentrations below 2 mM Si(OH)_4_, with soils typically containing Si(OH)_4_ concentrations of 0.1-0.6 mM (Epstein, 1994; Ma and Yamaji, 2006). Soils can therefore contain high levels of total Si, but low and potentially deficient levels of plant-available Si (Thorne et al., 2020).

Plants vary considerably in their abilities to accumulate Si (Hodson et al., 2005), with rice accumulating up to 10% Si by dry mass (Epstein, 1994). Seven of the ten crops with the highest global production are known Si accumulators, including rice, wheat, barley and maize (Guntzer et al., 2012). The ability to accumulate different levels of Si has been associated with Si transporters initially identified in rice, with several homologs being discovered in other species more recently (Mitani-Ueno and Ma, 2021). In rice, a combination of passive and active efflux transporters transport Si from the soil through the roots into the xylem (Ma et al., 2006, 2007; Huang et al., 2022). From here, it is translocated to the shoot via transpiration before being unloaded and deposited as hydrated silica (SiO_2_·nH_2_O) at target sites, again through a combination of passive and active transporters (Yamaji et al., 2008; Mitani-Ueno et al., 2023). This form of Si is immobile and cannot be redistributed once deposited (Epstein, 1994), though Si can be directed to specific sites within the plant (Thorne et al., 2023).

In addition to this strong link between Si uptake and transpiration, Si has been proposed to improve the resilience of plants to drought stress through alterations in gas exchange, with several studies showing that Si fertilization can impact both transpiration and stomatal conductance (g_s_). For example, Si treatment has been observed to increase (Hattori et al., 2005; Ashfaq et al., 2024), have no impact on (Johnson et al., 2022) or decrease (Gao et al., 2005; 2006) g_s_ and transpiration in drought-stressed plants in a variety of species. A recent meta-analysis of 34 studies (excluding rice) explored the role of Si in plant water movement; here, the authors found that Si had no consistent pattern on these parameters in unstressed plants, but significantly increased g_s_ (although not transpiration) in drought-stressed plants (Cooke and Carey, 2023).

One mechanism through which Si has been proposed to impact transpiration is by altering root hydraulic conductance through the regulation of aquaporin activity (Liu et al., 2015). Another potential mechanism is the sub-cuticular deposition of Si, which has been suggested to reduce transpiration from the cuticle by acting as a physical barrier, although the contribution this could make to total leaf transpiration is small compared to water loss via the stomata (Agarie et al., 1998). Other studies have suggested that Si deposited in the cell walls of stomatal complexes may play a role in altering the gas exchange of Si-treated plants. For example, Ueno and Agarie (2005) proposed that silica deficiency in the cell walls of rice stomatal complexes could increase evaporative water loss from the epidermis, thus disrupting the generation of turgor pressure required for stomatal opening/closure. More recent research in tall fescue (*Festuca arundinacea*) has suggested that the deposition of Si in stomatal guard cells could promote increased stomatal sensitivity, mediated by K^+^ transporters (Vandegeer et al., 2021).

Given the impact of Si treatment on gas exchange varies between different experiments, the specific mechanistic steps that occur between Si fertilizer application and an altered gas exchange response remain poorly understood (Coskun et al., 2016). Furthermore, it is unclear how alterations in gas exchange can lead to improvements in drought resilience. Despite these complexities, however, many studies do report significant biomass and/or yield increases with Si treatment under drought stress in wheat (e.g. Bukhari et al., 2021; Ayed et al., 2022; Johnson et al., 2022), though some do not (Thorne et al., 2021), and responses are often genotype-specific (Thorne et al., 2021; Ayed et al., 2022; Christian et al., 2023).

This study investigated mechanisms underpinning this variation by quantifying the impact of Si treatment on wheat water use and drought resilience. To do this, we first assessed the impact of Si treatment on two wheat genotypes under well-watered and drought conditions. We then explored potential factors driving the observed genotype-specific responses to Si treatment, focusing on differences in stomatal density. Finally, we investigated how the type and duration of drought (watering regime) affected the ability of Si to provide drought alleviation.

## 2 Materials and methods

### 2.1 Plant material, experimental design and growth conditions

To study the impact of Si treatment on wheat (*Triticum aestivum*) water use and drought resilience under different types of drought stress, three different experimental pipelines were followed.

Whilst watering regime varied between experiments, plants were always subjected to one of four treatments: (a) 0 mM Si, well-watered; (b) 0 mM Si, drought; (c) 1.8 mM Si, well-watered; or (d) 1.8 mM Si, drought. The 1.8 mM Si treatment was implemented by adding 1.8 mM sodium metasilicate (Na_2_SiO_3_·H_2_O) to compost and 3.6 mM sodium chloride (NaCl) was used for the 0 mM Si treatment (to balance the Na^+^ ions).

Experiments 1 and 3 were carried out in controlled environment chambers (16-hour/20°C day, 8-hour/16°C night, 60% relative humidity, 400 μmols^-1^m^-2^ PPFD). Experiment 2 was carried out in a controlled glasshouse (16-hour/20°C day, 8-hour/16°C night, 50% relative humidity).

#### 2.1.1 Experiment 1 (Exp1): short-term severe drought stress imposition

To investigate the impact of Si treatment on wheat water use and drought resilience, two high Si accumulating genotypes (H3 and H5) from the YoGI biodiversity panel were selected (Thorne et al., 2021; Barratt et al., 2023). High Si accumulating genotypes were studied to maximize the differences in plant Si concentration between Si treatments.

Germinated seedlings were transplanted into 11 x 11 x 12 cm pots containing 400 g 6:1 M3 compost (Levington):perlite supplemented with slow-release fertilizer (Osmocote Exact Standard 5-6). The growth substrate had a plant-available Si concentration of 0.23 ± 0.04 mM, as determined using the molybdate method following CaCl_2_ extraction (Sauer et al., 2006; Liang et al., 2015). Pots were arranged within trays containing 3 plants per genotype. For each genotype and treatment, n=9 (apart from H5 in treatment (b) where n=8). Trays were supplied with equal amounts of 0 mM or 1.8 mM Si solution 3 times a week and kept well-watered with additional water if needed. To implement drought stress, Si treatments and additional watering were withheld for 11 days around the booting stage of development (weeks 8/9) for droughted plants. Si treatments stopped for well-watered plants during this period, but water was still supplied. After 11 days, pre-drought Si and watering regimes were resumed. Plants were then grown to yield.

#### 2.1.2 Experiment 2 (Exp2): drought treatment of low stomatal density wheat

To explore the role of stomatal density in the response of wheat to Si treatment and drought stress, a modern cultivar (Fielder) was grown alongside a reduced stomatal density Fielder line (*TaEPF1OE*). The phenotype in *TaEPF1OE* is achieved by overexpressing *EPF1 (EPIDERMAL PATTERNING FACTOR 1)*, which encodes a negative regulator of stomatal development (Dunn et al., 2019).

Germinated seedlings were transplanted into 11 x 11 x 12 cm pots containing 450 g F2+S compost (Levington; plant-available Si concentration of 0.13 ± 0.04 mM) supplemented with osmocote and thinned to 1 plant per pot after 12 days. Pots were arranged within trays containing 2 plants per genotype. For each genotype and treatment, n=10. Si treatments and drought stress were carried out as described in Exp1, with the exceptions of drought stress being implemented during weeks 7/8 (to align with the booting stage of the development) and the drought stress lasting 7 days (as plants dried out more quickly). Plants were then grown to yield.

#### 2.1.3 Experiment 3 (Exp3): long-term drought stress imposition

Following significant results in Exp1 and Exp2, H5 and Fielder were subjected to a differing water regime. This experiment aimed to test if their responses to Si treatment under drought stress are specific to the watering regime.

Germinated seedlings were transplanted into 9 x 9 x 10 cm pots containing 270 g F2+S compost supplemented with osmocote and thinned to 1 plant per pot after 1 week. Pots were arranged within trays containing 4 plants per genotype. For each genotype and treatment, n=8. This experiment aimed to subject plants to contrasting relative Soil Water Contents, rSWC (80% *vs* 20% rSWC). 100% rSWC was determined by fully soaking 6 pots containing compost (but no plants), before draining and weighing. These pots were then dried at 60°C until constant mass was achieved (0% rSWC). Target masses for experimental pots were then calculated using the mean 100% and 0% rSWC values and the following equations:


$$\begin{array}{c} 80\%\;\text{rSWC}=\mathrm{pot}\;\mathrm{mass}+0\%\;\mathrm{rSWC}+0.8(100\%\;\mathrm{rSWC}\\ -0\%\;\mathrm{rSWC}) \end{array}$$

$$\begin{array}{c} 20\%\;\mathrm{rSWC}=\mathrm{pot}\;\mathrm{mass}+0\%\;\mathrm{rSWC}+0.2(100\%\;\mathrm{rSWC}\\ -0\%\;\mathrm{rSWC}) \end{array}$$

Plants were initially well-watered 3 times a week with equal amounts of 0 mM or 1.8 mM Si solutions. After 2 weeks, watering was stopped until pots reached their target masses. Prior to all pots reaching their target masses, pots that had dropped below their target masses were watered with appropriate amounts of water. When all plants had reached their target masses, equal amounts of 0 mM or 1.8 mM Si solutions were added to each pot, with the amount added being the minimum mass difference observed between a pot’s mass and its target mass. Any mass difference was made up by adding water. Pots were weighed and Si treated/watered 3 times a week.

### 2.2 Water use during drought

During the drought stress implemented in Exp1 and Exp2, droughted plants were weighed daily at 15:00. Normalized pot masses were calculated by dividing each pot’s daily mass by its mass at the start of the drought period. Normalized water use during drought was determined by subtracting the final day’s normalized pot mass from the first day’s normalized pot mass. In Exp3, pots were weighed 3 times per week during the drought period from 15:00. Total water added during drought was calculated by summing the pot mass difference between each time point measured during drought.

### 2.3 Stomatal density measurements

Dental resin was used to make negative impressions of abaxial and adaxial surfaces at the mid-point of recently fully expanded leaves (Exp1: leaf 6, week 6; Exp2: leaf 5, weeks 5-6; and Exp3: leaf 6 for H5, leaf 5 for Fielder, week 6). Resin impressions were coated with clear nail varnish. Once dried, nail varnish impressions were mounted on slides and visualized at 10X magnification using a Brunel n300-M light microscope equipped with a Prior ES10ZE Focus Controller and Moticam 5 camera. 5 images were taken per slide. ImageJ (Schneider et al., 2012) was used to attain the mean abaxial, adaxial and total stomatal densities (as stomata mm^-2^) for each plant.

### 2.4 Gas exchange measurements

#### 2.4.1 Light shifts

For Exp1 and Exp2, the responses of stomatal conductance (g_s_) and photosynthetic assimilation (A) to shifts in light intensity were recorded using two portable infrared gas analyzers (IRGAs, LI-6800, LI-COR, USA). The mid-section of recently fully expanded leaves was used (Exp1: leaf 6, week 6; and Exp2: leaf 5, weeks 5-6). Measurements were carried out at 400 ppm CO_2_, with a leaf temperature of 20°C, a fan speed of 10000 rpm, an air flow of 400 μmols^-1^ and a relative humidity of 60% (Exp1) or 50% (Exp2). IRGAs were matched a few minutes after leaves were clamped into the chamber. The light source was first set to 100 PAR and plants were allowed to stabilize. The light shift protocol involved the following steps: 5 minutes at 100 PAR; 90 (Exp1) or 60 (Exp2) minutes at 1000 PAR (opening response); and 90 (Exp1) or 60 (Exp2) minutes at 100 PAR (closing response). A and g_s_ were recorded every minute. Data points recorded at the exact time of the light shifts were removed, as were any negative g_s_ values. The initial g_s_ (initial g_min_) was recorded as the final data point during the first 5 minutes at 100 PAR. g_min_ for the closing response (closing g_min_) was extracted as the minimum g_s_ reached during the final 100 PAR step.

#### 2.4.2 Steady-state

In Exp3, steady-state gas exchange measurements were carried out using three portable infrared gas analyzers (IRGAs, LI-6800, LI-COR, USA) during week 6. The mid-section of the most recently fully expanded leaf was used (leaf 6 for H5, leaf 5 for Fielder). Measurements were carried out at the same conditions as Exp1, but with the light source set to 400 PAR. Plants were allowed to stabilize before A and g_s_ were recorded every 30 seconds for 5 minutes. These data points were averaged for each plant to calculate mean steady-state values of A, g_s_ and intrinsic water use efficiency (iWUE, A/g_s_).

### 2.5 Si measurements

To measure Si concentration, several leaves from each plant were collected and fully dried at 60°C (Exp1: week 11; Exp2: week 7; and Exp3: week 6). As described previously (Reidinger et al., 2012), dried leaf material was ground using a TissueLyser II (Qiagen, Manchester, UK) and pressed into pellets using a manual hydraulic press at 10 tons with a 13 mm die (Specac, Orpington, UK). A portable X-ray fluorescence (P-XRF) instrument (Nitron XL3t900 GOLDD analyzer, Thermo Scientific, Winchester UK) in a test stand (SmartStand, Thermo Scientific) was used to measure Si concentration (%). Once calibrated (using Si-spiked synthetic methyl cellulose, Sigma-Aldrich, product no. 274429) and validated (using Certified Reference Materials of NCS DC73349 ‘Bush, branches and leaves’ obtained from China National Analysis Center for Iron and Steel), measurements were performed under a helium atmosphere to avoid signal loss by air absorption. Both sides of each pellet were measured and readings were averaged to calculate the mean Si concentration (%) of each pellet.

### 2.6 Yield measurements

For Exp1 and Exp2, yield measurements were carried out when plants were fully dried. For each plant, the total aboveground dried biomass and total seed mass was measured. In Exp3, plants were harvested at the end of week 6, fully dried at 60°C and weighed for total aboveground dried biomass.

### 2.7 Statistical analyses

All statistical analyses were performed using R software version 4.4.1 (R Core Team, 2024) and figures were produced using ggplot2 (Wickham, 2016). All data are presented as mean ± standard error unless otherwise stated. Two-way or three-way ANOVAs using the aov() function were performed to test the effect of genotype, Si treatment and drought treatment on various parameters. Data normality was checked using Q-Q plots and Shapiro-Wilk tests (shapiro.test() function). Levene’s test (leveneTest() function) was used to check for equal variance (Fox and Weisberg, 2019). If data were transformed to meet ANOVA assumptions, or did not satisfy ANOVA assumptions, this is noted in the relevant **Supplementary Tables**. The emmeans() function (Lenth, 2024) was used for *post-hoc* comparisons, testing for statistically significant pairwise differences in parameter means between the 0 mM and 1.8 mM Si treatments for each genotype/drought treatment.

## 3 Results

### 3.1 High Si accumulating genotypes varied significantly in stomatal density and in their responses to Si treatment and drought stress

**Figure 1:**
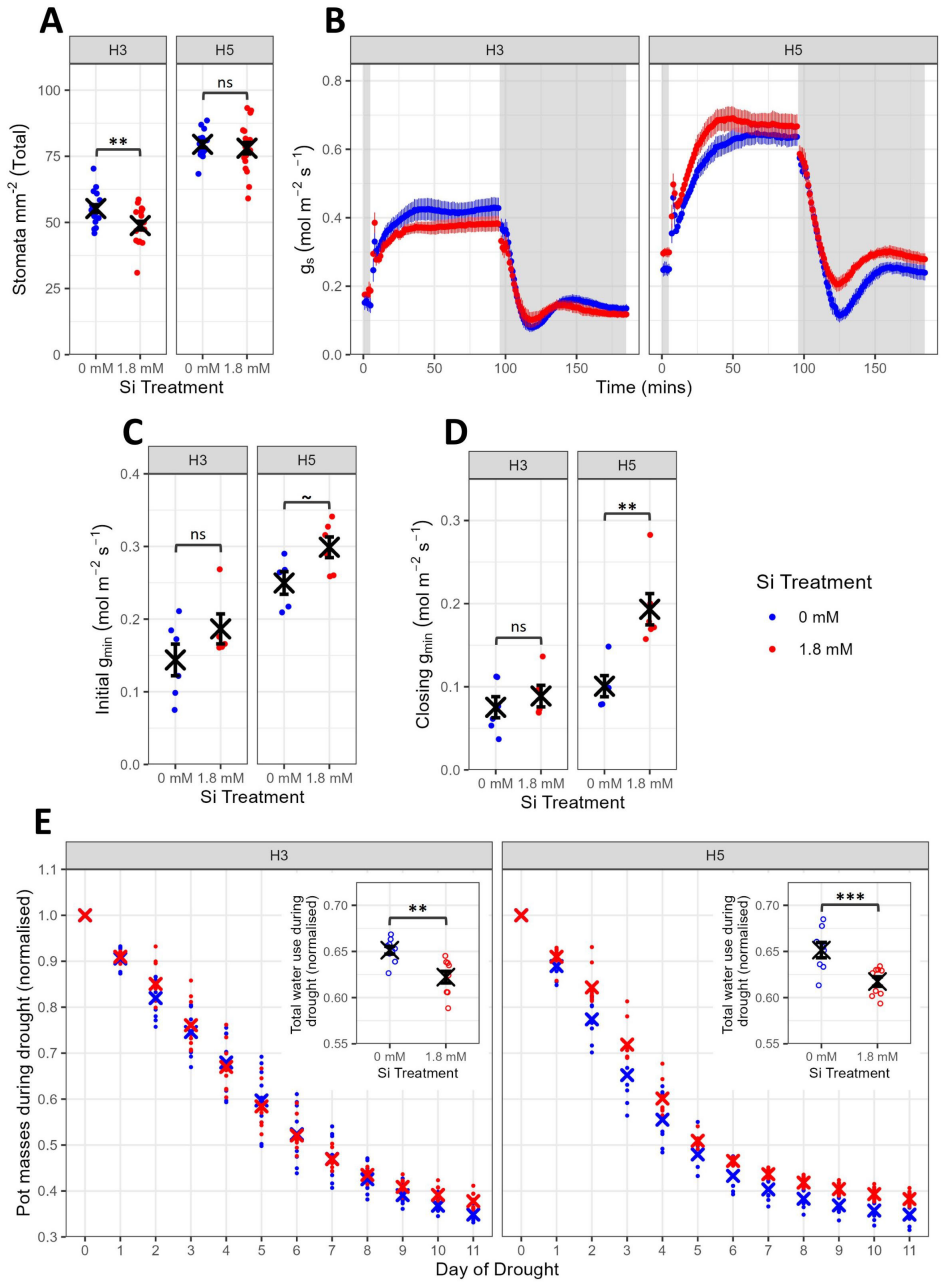
Impact of Si on high Si accumulating wheat genotypes H3 and H5 (Exp1). **(A)** Stomatal density (total of abaxial and adaxial) of leaf 6 on 6-week-old plants (n=17-18). **(B)** Response of g_s_ to shifts in light intensity, measured by infra-red gas analysis of leaf 6 on 6-week-old plants (n=5-6). Grey-shaded background represents 100 PAR (low light) and white background 1000 PAR (high light). **(C)** Initial g_min_, the final data point during the first 5 minutes after readings stabilised at 100 PAR, extracted from panel B (n=5-6). **(D)** Closing g_min_, the minimum g_s_ reached during the final 100 PAR step extracted from panel B (n=5-6). **(E)** Normalised daily pot masses during drought, calculated by dividing each pot mass by its mass at the start of the drought period (n=8-9). Insets show total water use during drought, calculated by subtracting the final day normalised pot mass from the first day normalised pot mass (n=8-9). Blue points represent 0 mM Si treatment, red points represent 1.8 mM Si treatment. Mean values ± SE are shown. The emmeans() package in R was used to test for statistically significant pairwise differences in parameter means between the 0 mM and 1.8 mM Si treatments for each genotype. ns non-significant, P<0.1 ~, P<0.01 **, P<0.001 ***.

To investigate the impact of Si treatment on wheat water use and drought resilience, two high Si accumulating genotypes (H3 and H5) were studied. To check whether the two genotypes displayed any intrinsic differences in stomatal characteristics which might influence the water-use response to exogenous Si, we first measured stomatal density, both with and without supplemental Si. Total stomatal density (**Figure 1A**) varied significantly between genotypes (F_1,67_=256.0, P<0.0001) and Si treatments (F_1,67_=5.7, P=0.0201). These comparisons were also significant for abaxial stomatal density, although only genotype had a significant impact on the adaxial surface values (**Supplementary Figures 1A, B**; **Supplementary Table 1**). Total basal stomatal density was approximately 1.5 times higher in H5 than in H3, with Si treatment significantly reducing total stomatal density in H3 (t_67_=2.7, P=0.0078) but having no impact on H5 (t_67_=0.6, P=0.544).

To find out whether these differences in stomatal density were reflected in stomatal conductance (g_s_), infra-red gas exchange analysis (IRGA) was performed (**Figure 1B**). In accordance with the observed differences in stomatal densities, the genotype H5 reached much higher levels of g_s_ than H3 during light shift experiments in the IRGA leaf chamber. In the presence of 1.8 mM Si, the H3 plants showed a slightly reduced g_s_ compared to plants grown with no Si supplementation, in line with the observed decrease in stomatal density. In contrast, there was an increase in g_s_ in the H5 plants supplemented with Si, particularly under low light levels, despite there being no observable shift in stomatal density. To quantify this, we extracted the parameter g_min_ from both the start of the light shifts (initial g_min_) and during the closing response (closing g_min_). Interestingly, g_min_ varied significantly with genotype and Si treatment in both the initial measurement (**Figure 1C**, G: F_1,18_=37.7, P<0.0001; Si: F_1,18_=6.1, p=0.0233) and in the closing response (**Figure 1D**, G: F_1,18_=20.3, P=0.000275; Si: F_1,18_=10.2 P=0.00498). *Post-hoc* testing highlighted a non-significant trend for increased initial g_min_ (t_18_=-1.9, P=0.0771) and a significant increase in g_min_ for H5 closing after Si treatment (t_18_=-3.5, P=0.0028). This suggests that Si treatment resulted in a reduction in the ability of stomata to fully close under low light conditions in a genotype-specific manner. In contrast to g_s_, minimal differences were observed between genotypes and Si treatments for the rate of carbon assimilation, A (**Supplementary Figure 1C**).

To investigate whether there were differences in overall water-use during drought between plants supplied with or without 1.8 mM Si, pot masses were monitored over time subsequent to the initiation of drought (withholding of water) (**Figure 1E**). Pot masses decreased considerably during the 11-day drought period for both genotypes irrespective of treatment, with Si treatment significantly reducing the normalized total water use during drought (**Figure 1E**, F_1,31_=27.0, P<0.0001). Si-treated plants used around 5% less water than control plants in both H3 (t_31_=3.4, P=0.0016) and H5 (t_31_=3.9, P=0.0005).

To assess the accumulation of Si during the experiment, XRF measurements were carried out on leaves sampled post-drought. These results showed that Si supplementation significantly increased leaf Si concentration (**Supplementary Figure 1D**, F_1,63_=204.1, P<0.0001), with Si concentration varying significantly between genotypes (F_1,63_=11.4, P=0.00126).

**Figure 2:**
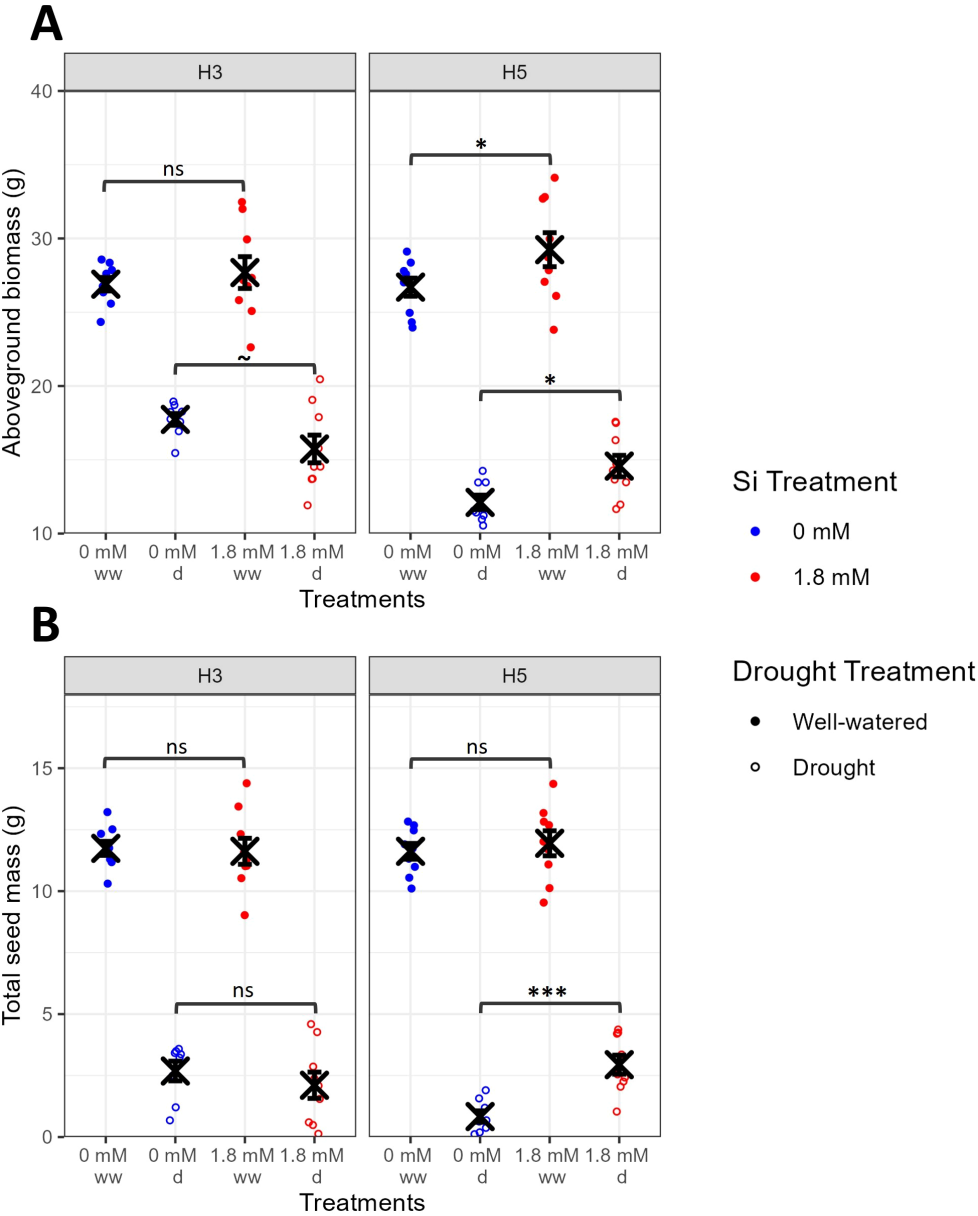
Impact of Si treatment and drought stress on yield measurements for high Si accumulating genotypes H3 and H5 (Exp1). **(A)** Aboveground biomass (n=8-9). **(B)** Total seed mass (n=8-9). Blue points represent 0 mM Si treatment, red points represent 1.8 mM Si treatment. Closed points represent well-watered plants, open points represent droughted plants. Mean values ± SE are shown. The emmeans() package in R was used to test for statistically significant pairwise differences in parameter means between the 0 mM and 1.8 mM Si treatments for each genotype and drought treatment. ns non-significant, P<0.1 ~, P<0.05 *, P<0.001 ***.

There were significant differences in aboveground biomass between genotypes (**Figure 2A**, F_1,62_=4.8, P=0.0322), with drought treatment causing significant reductions in biomass (F_1,62_=499.2, P<0.0001). Significant interaction terms (**Supplementary Table 2**) suggest that the impact of Si treatment and drought stress on aboveground biomass varied between genotypes, with Si-treated H5 accumulating significantly increased biomass in both well-watered (t_62_=-2.3, P=0.0260) and drought (t_62_=-2.1, P=0.0357) conditions but Si treatment having no significant impact on the biomass of H3.

Total seed mass was significantly and drastically reduced by drought treatment (**Figure 2B**, F_1,62_=1041.7, P<0.0001). Si treatment acted significantly differently on the two genotypes (G x Si: F_1,62_=6.5, P=0.0131). In H5, Si treatment significantly alleviated the impact of drought on seed yield, with total seed mass being 3.5-fold higher for plants supplied with Si under drought stress relative to plants droughted and not supplied with Si (t_62_=-3.5, P=0.0009). However, there was no impact of Si on H5 under well-watered conditions or on H3 under well-watered or drought conditions (**Supplementary Table 4**).

### 3.2 Altering stomatal density impacted the response of g_s_ to Si treatment but had limited impact on other plant responses to Si treatment and drought stress

**Figure 3:**
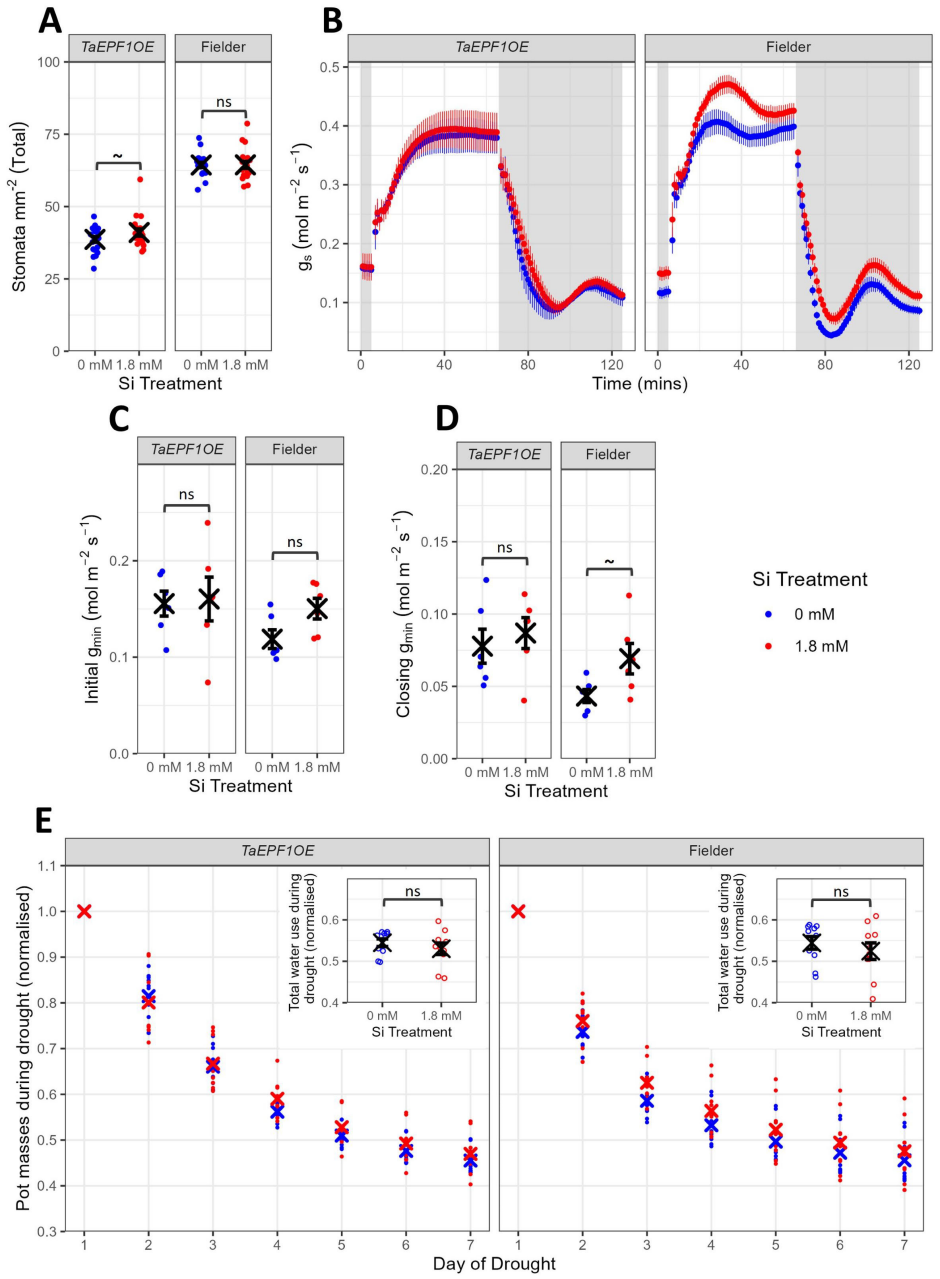
Impact of Si on reduced stomatal density mutant (*TaEPF1OE*) and non-transgenic Fielder control genotype (Exp2). **(A)** Total stomatal density of leaf 5 on 5-6-week-old plants (n=20). **(B)** Response of g_s_ to shifts in light intensity, measured using infra-red gas analysis on leaf 5 during weeks 5-6 (n=6). Grey-shaded background represents 100 PAR (low light) and white background 1000 PAR (high light). **(C)** Initial g_min_ (n=6). **(D)** Closing g_min_ (n=6). **(E)** Normalised daily pot masses during drought. Insets show total water use during drought (n=10). Blue points represent 0 mM Si treatment, red points represent 1.8 mM Si treatment. Mean values ± SE are shown. The emmeans() package in R was used to test for statistically significant pairwise differences in parameter means between the 0 mM and 1.8 mM Si treatments for each genotype. ns non-significant, P<0.1 ~.

**Figure 4:**
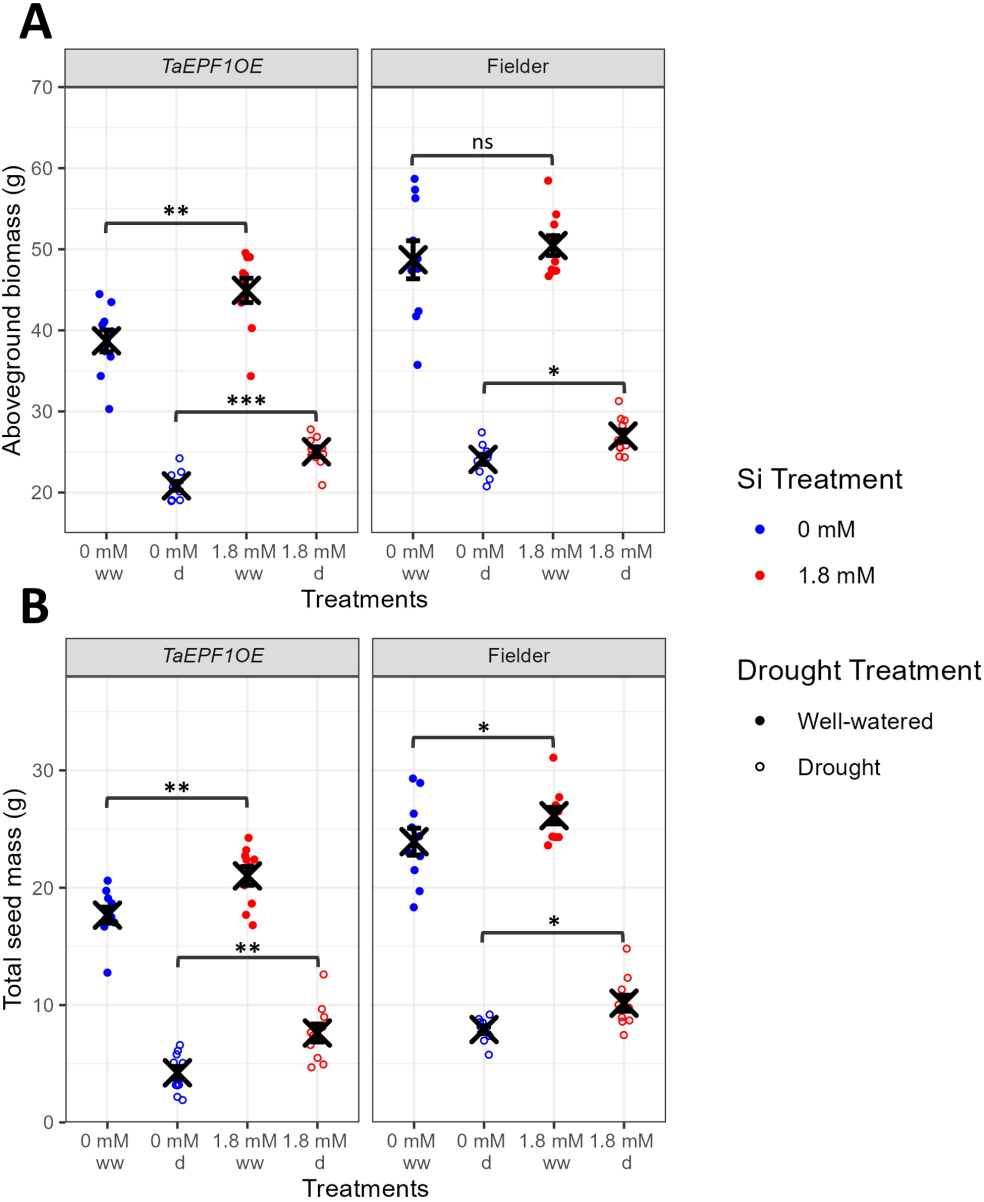
Impact of Si treatment and drought stress on yield measurements for reduced stomatal density mutant (*TaEPF1OE*) and control Fielder genotype (Exp2). **(A)** Aboveground biomass (n=10). **(B)** Total seed mass (n=10). Blue points represent 0 mM Si treatment, red points represent 1.8 mM Si treatment. Closed points represent well-watered plants, open points represent droughted plants. Mean values ± SE are shown. The emmeans() package in R was used to test for statistically significant pairwise differences in parameter means between the 0 mM and 1.8 mM Si treatments for each genotype and drought treatment. ns non-significant, P<0.05 *, P<0.01 **, P<0.001 ***.

To investigate whether the differences in response to Si between H3 and H5 genotypes described above could be mechanistically driven by their intrinsic differences in stomatal density (**Figure 1A**), we performed a series of experiments using a transgenic wheat line engineered to have a lower stomatal density, *TaEPF1OE* (Dunn et al., 2019). Since this line had been engineered in the Fielder background, a non-transgenic Fielder line was used as the appropriate control for these experiments. A similar set of experiments (as described for H3 and H5) was performed, with the results shown in **Figures 3** and **4**.

As expected, the *TaEPF1OE* line had a significantly lower stomatal density compared to Fielder (**Figure 3A**, F_1,76_=472.2, P<0.0001). This decrease in stomatal density was observed on both the abaxial and adaxial surfaces of the leaf (**Supplementary Figures 2A, B**; **Supplementary Table 5**). After Si treatment, there were minimal significant changes in total stomatal density in the *TaEPF1OE* line or the background Fielder line (**Figure 3A**; **Supplementary Tables 5**, **7**). This contrasted with the significant decrease in stomatal density observed in H3 after Si treatment but was in line with results for H5 (**Figure 1A**).

Minimal differences in g_s_ were observed between Si treatments in the low stomatal density line *TaEPF1OE* under IRGA light shifts (**Figure 3B**). However, increases in g_s_ in Si-treated plants were observed for the non-transgenic Fielder line under both 1000 and 100 PAR light, similar to the changes observed in H5 (**Figure 1B**). The impact of genotype and Si treatment on g_min_ varied between the initial g_min_ (**Figure 3C**, G: F_1,20_=2.5, P=0.132; Si: F_1,20_=1.5, P=0.236) and closing g_min_ (**Figure 3D**, G: F_1,20_=7.1, P=0.0150; Si: F_1,20_=3.2, P=0.0896) for both the *TaEPF1OE* and Fielder lines. As seen for H5 (**Figure 1D**), Si supplementation of Fielder increased closing g_min_, although this was not quite significant (t_20_=-1.9, P=0.0769). Si treatment and genotype had no impact on rate of carbon assimilation, A, under IRGA light shifts for these lines (**Supplementary Figure 2C**).

Si treatment significantly increased Si concentration in pre-drought plants for both *TaEPF1OE* and Fielder lines (**Supplementary Figure 2D**, F_1,76_=824.8, P<0.0001). Notably, genotype had no significant impact on Si concentration (F_1,76_=0.8, P=0.377), suggesting that the reduction in stomatal density in *TaEPF1OE* plants had no impact on Si uptake.

In contrast to the H3 and H5 genotypes, Si treatment had no significant impact on normalized total water use during drought for both *TaEPF1OE* and Fielder lines (**Figure 3E**, F_1,36_=1.3, P=0.259), although there was a trend for decreased water use during drought following Si treatment.

Drought stress significantly reduced aboveground biomass (**Figure 4A**, F_1,72_=769.0, P<0.0001) and total seed mass (**Figure 4B**, F_1,72_=794.4, P<0.0001) in both *TaEPF1OE* and Fielder lines. In general, these yield parameters were significantly increased with Si treatment relative to control and were significantly higher in Fielder compared to *TaEPF1OE* (**Supplementary Tables 6**, **8**). In genotype-specific Si treatment comparisons, 1.8 mM Si treatment significantly increased total seed mass for both genotypes under both well-watered (*TaEPF1OE*: +18.6%, t_72_=-3.2, P=0.0020; Fielder: +9.2%, t_72_=-2.1, P=0.0371) and droughted (*TaEPF1OE*: +80.1%, t_72_=-3.2, P=0.0018; Fielder: +28.8%, t_72_=-2.2, P=0.0348) conditions. This suggests that Si supplementation can be beneficial under non-stressed conditions as well as during stress, but this observation is likely specific to genotype and growth conditions.

### 3.3 The responses of H5 and Fielder lines to Si treatment depend upon the watering regime

**Figure 5:**
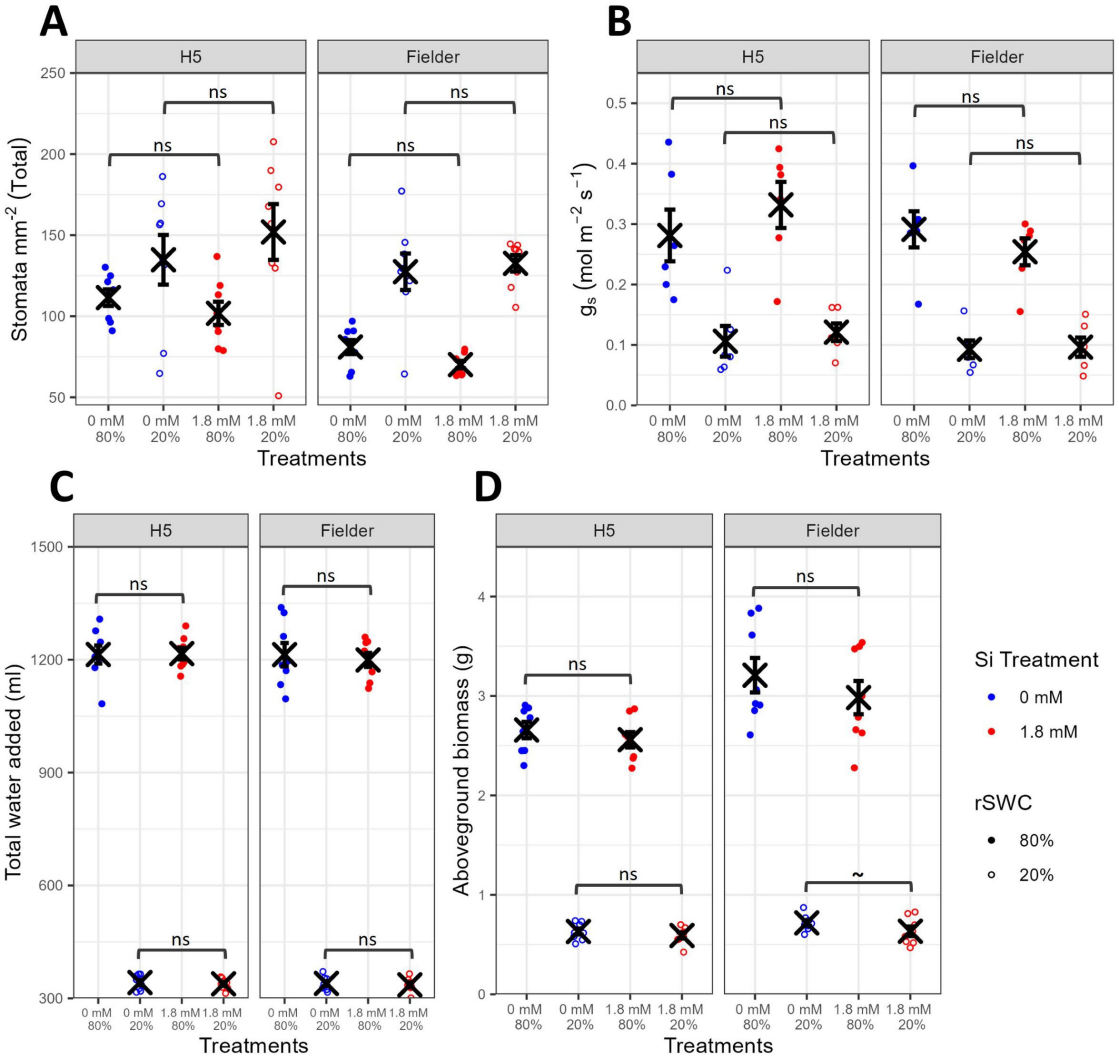
Impact of Si treatment and rSWC on H5 and Fielder genotypes (Exp3). **(A)** Total stomatal density of leaf 6 for H5, leaf 5 for Fielder on 6-week-old plants (n=8). **(B)** Steady-state g_s,_ measured using infra-red gas analysis on leaf 6 for H5, leaf 5 for Fielder during week 6 (n=6). **(C)** Total water volume added to each pot to maintain 80% or 20% rSWC; i.e. total water use during the drought period (n=8). **(D)** Aboveground dry biomass of plants harvested at the end of week 6 (n=8). Blue points represent 0 mM Si treatment, red points represent 1.8 mM Si treatment. Closed points represent 80% rSWC treatment, open points represent 20% rSWC treatment. Mean values ± SE are shown. The emmeans() package in R was used to test for statistically significant pairwise differences in parameter means between the 0 mM and 1.8 mM Si treatments for each genotype and rSWC treatment. ns non-significant, P<0.1 ~.

It was hypothesized that the observed increases in g_min_ with Si treatment in H5 (**Figure 1B**) and Fielder (**Figure 3B**) during IRGA light shifts could help improve wheat drought resilience under a more prolonged drought stress by allowing plants to maintain g_s_ and thus A. We investigated this by subjecting H5 and Fielder to contrasting relative Soil Water Contents, rSWC (80% *vs* 20% rSWC), throughout vegetative growth. Given these genotypes both exhibited significant increases in biomass and total seed mass under short-term severe drought stress with Si treatment (**Figures 2**, **4**), we further hypothesized that Si-treated plants would show increases in aboveground biomass under 20% rSWC. In contradiction to our hypotheses, most parameters measured were only significantly impacted by genotype and/or rSWC, with Si treatment having minimal significant impacts (**Figure 5**, **Supplementary Figure 3**; **Supplementary Tables 9**, **10**).

Total stomatal density (**Figure 5A**) varied significantly between the H5 and Fielder lines (F_1,56_=10.0, P=0.00247), with the 20% rSWC treatment significantly increasing stomatal density compared to the 80% rSWC treatment (F_1,56_=42.4, P<0.0001). The same observations were also made for the abaxial and adaxial surfaces (**Supplementary Figures 3A, B**; **Supplementary Table 9**).

Steady-state IRGA measurements showed that Si treatment had no significant impact on g_s_, A or iWUE under these conditions for these two genotypes (**Figure 5B**, **Supplementary Figures 3C, D**; **Supplementary Table 9**). In contrast, reducing rSWC significantly reduced g_s_ (F_1,40_=91.8, P<0.0001) and A (F_1,40_=47.7, P<0.0001), whilst significantly increasing iWUE (F_1,40_=116.8, P<0.0001). In accordance with these steady-state g_s_ and iWUE results, total water added to plants (i.e. water used during the drought period, **Figure 5C**) was significantly lower in the 20% rSWC plants compared to the 80% rSWC plants (F_1,56_=8993.6, P<0.0001) and was not significantly impacted by Si treatment (F_1,56_=0.3, P=0.604).

Si concentration (**Supplementary Figure 3E**) was significantly impacted by genotype (F_1,56_=6.1, P=0.0169), Si treatment (F_1,56_=176.5, P<0.0001) and rSWC (F_1,56_=43.1, P<0.0001), with the interaction between Si and rSWC also being significant (F_1,56_=16.5, P=0.000156).

Plants grown at low rSWC showed significantly reduced aboveground biomasses compared to well-watered plants (**Figure 5D**, F_1,56_=1744.2, P<0.0001). Si treatment also significantly impacted aboveground biomass (F_1,56_=4.9, P=0.0304). However, in contrast to the previous water withholding experiments, here the 1.8 mM Si treatment tended to reduce total aboveground biomass rather than increase it, although *post-hoc* testing showed these trends to be non-significant (H5 80%: -3.76%, t_56_=0.5, P=0.606; H5 20%: -6.34%, t_56_=1.0, P=0.346; Fielder 80%: -7.17%, t_56_=1.0, P=0.305 and Fielder 20%: -11.7%, t_56_=1.9, P=0.0579). This suggests that the impact of Si on wheat yields under drought stress is strongly linked to watering regime.

## 4 Discussion

In this study, we aimed to assess the impact of Si treatment on wheat water use and drought resilience under different watering regimes. By comparing several different genotypes and contrasting watering regimes, we found that the impact of Si treatment on wheat water use and drought resilience is strongly affected by genotype and watering regime. We also explored the impact of manipulating stomatal density on the wheat response to Si treatment to determine whether differences in stomatal density could account for genotype-specific responses. Our results suggest that stomatal density alone cannot be responsible for genotype-specific yield responses to Si treatment, but that stomatal density could play a key role in the impact of Si treatment on g_s_ and stomatal function.

To explore the role of Si in wheat water use, we first grew two high Si accumulating genotypes (H3 and H5) under 0 mM or 1.8 mM Si treatments. A recent meta-analysis of 34 studies (excluding rice) found that supplemental Si had no significant impact or consistent pattern on transpiration or g_s_ in unstressed plants (Cooke and Carey, 2023). We therefore hypothesized that under well-watered conditions, plants would exhibit minimal g_s_ differences. This appeared to be correct for H3 during IRGA-induced light shifts (**Figure 1B**). However, in H5 we observed considerable increases in g_s_ under well-watered conditions with Si, particularly in closing g_min_ (**Figure 1D**). This suggests that Si may be reducing the ability of stomata to fully close under low light conditions in a genotype-specific manner. This could be due to the deposition of Si in the cell walls of stomatal guard cells, an observation that has been made in several species (Sakai and Thom, 1979; Kim et al., 2002; Motomura et al., 2004; Ueno and Agarie, 2005; Law and Exley, 2011; Pierantoni et al., 2017). Our findings contrast with those of a study in tall fescue, where guard cell Si deposition was linked to a reduction in g_s_ in non-stressed plants and an increase in stomatal sensitivity (Vandegeer et al., 2021), suggesting that responses may be species-specific.

To test the role of Si treatment in these wheat genotypes under drought stress, we withheld water at the booting stage of development. In droughted plants, Si has been shown to increase (Hattori et al., 2005; Cooke and Carey, 2023; Ashfaq et al., 2024), decrease (Gao et al., 2005; 2006) or have no impact (Johnson et al., 2022) on transpiration and/or g_s_, or even simultaneously increase transpiration whilst decreasing g_s_ (Chen et al., 2011). Here, we found that Si-treated plants used significantly less water than control plants during stress (**Figure 1E**), corroborating reports that Si can act to reduce g_s_ and transpiration under drought stress. However, this reduction in water use did not necessarily lead to improved drought resilience. Indeed, the impact of Si on yields varied considerably between H3 and H5, with significant Si-associated yield increases under drought stress for H5 and non-significant reductions in yields for H3 (**Figure 2**). This supports several recent studies where the impact of Si on wheat yields under drought stress has been shown to be genotype-specific (Thorne et al., 2021; Ayed et al., 2022; Christian et al., 2023).

To investigate whether the differing response to Si in H3 and H5 could be linked to their contrasting stomatal densities (**Figure 1A**), we compared the responses of a reduced stomatal density transgenic Fielder line (*TaEPF1OE*) and a Fielder control to Si treatment. We reasoned that, if the different responses of H3 and H5 to Si treatment were reflected in the experiments involving *TaEPF1OE* and Fielder, higher stomatal densities could be a key factor in driving beneficial responses to Si treatment. In the IRGA-induced light shifts, *TaEPF1OE* and Fielder (**Figures 3B–D**) behaved similarly to H3 and H5 (**Figures 1B–D**) respectively, suggesting that stomatal density plays an important role in the impact of Si treatment on g_s_ and stomatal function. However, aside from the differences in gas exchange, minimal differences were observed between the reduced stomatal density and background lines. This suggests that stomatal density alone cannot be responsible for genotype-specific responses to Si and, therefore, that additional factors must be involved. These findings highlight the need to identify mechanisms that can directly translate to improvements in yields.

Si uptake occurs both passively, driven by transpiration, and actively (reviewed by e.g. Ma and Yamaji, 2015; Kumar et al., 2017b; Mandlik et al., 2020; Mitani-Ueno and Ma, 2021); however, it remains unclear whether Si accumulation is limited by the rate of transpiration or the rate of active uptake. If Si is primarily taken up by passive processes, it could be expected that by reducing stomatal density, transpiration is reduced and thus Si concentration may also be reduced. However, our results show that significantly reducing stomatal density had no impact on leaf Si concentration (**Supplementary Figure 2D**). These data add to the existing literature that highlights the importance of active transport in Si uptake and deposition (Liang et al., 2006; Gocke et al., 2013; Kumar et al., 2017a; McLarnon et al., 2017).

The yield data from this second experiment show that Si treatment can significantly increase yields under both stressful and non-stressful conditions in some genotypes (**Figure 4**), contrasting with the idea, presented by Coskun et al. (2019), that Si does not promote plant growth *per se* but does protect against stress. Whilst the majority of literature demonstrates the benefit of Si fertilizers under stressful conditions (e.g. see meta-analyses by Cooke and Leishman, 2016; Johnson et al., 2024), some studies have observed benefits under non-stressful conditions (e.g. Camargo et al., 2021; Malik et al., 2025), although it is important to recognize that a truly stress-free environment rarely exists (Frew et al., 2018). Regardless, these findings suggest that even plants growing in relatively ‘non-stressed’ environments can still benefit from Si fertilization.

To test the role of watering regime on the wheat response to Si treatment, we subjected two genotypes (H5 and Fielder) that responded positively to Si under the water withholding drought stress to a prolonged reduced rSWC drought stress. It was hypothesized that the observed increases in g_s_ with Si in H5 (**Figure 1B**) and Fielder (**Figure 3B**) during IRGA light shifts could help improve wheat drought resilience under a more prolonged drought stress by allowing plants to maintain g_s_ and thus A. However, under this contrasting watering regime, Si had no significant impact on most of the parameters studied, including stomatal density, steady-state g_s_, iWUE and total water use during drought (**Figure 5**). In contrast to the increased yields under drought stress observed with Si in the first two experiments, here, we observed non-significant reductions in aboveground biomass with Si.

These differing results could be due to the altered Si treatment regime in the contrasting drought treatments, with plants in the rSWC experiment receiving less Si, and thus accumulating less Si overall, than plants in the water withholding experiments (**Supplementary Figures 1D**, **2D**, **3E**). Several studies have found that the benefits of Si fertilizer vary with the way in which Si is applied, e.g. the type of Si fertilizer (Thakral et al., 2024; Christian et al., 2025), the application rate and/or concentration (Johnson et al., 2022; Shamshiripour et al., 2022; Cheraghi et al., 2024; Foresti et al., 2024; Malik et al., 2025), or the lifecycle stage/timing of application(s) (Ma et al., 1989; Lavinsky et al., 2016). In our study, the contrasting watering regimes were implemented at different wheat lifecycle stages, either at booting (water withholding) or throughout vegetative growth (rSWC). In rice, Si is re-directed to the husks during flowering by the action of specific transporters (Yamaji and Ma, 2009; Yamaji et al., 2015), with significant grain yield penalties being observed if the hyper-accumulation of Si in the husk is prevented (Tamai and Ma, 2008). If this is the case in wheat, it might explain why Fielder and H5 responded more positively to Si fertilizers under drought stress at the booting stage of development than during vegetative growth.

The contrasting responses of H5 and Fielder to Si treatment under differing water regimes could also be linked to the potential costs of Si uptake. A recent study proposed the existence of commonly ignored direct costs associated with Si accumulation, specifically highlighting examples where increased leaf Si has been negatively correlated with growth rate (de Tombeur et al., 2023). In our experiments, perhaps the cost of accumulating Si in leaf tissue under prolonged reduced rSWC was too great a proportion of diminished resources, leading to slight reductions in biomass with Si. The observed biomass responses in our study may also relate to inherent differences in a plant’s response to contrasting drought stresses. For example, the priority may be to rapidly close stomata under a short-term drought stress and then minimize overall transpiration in the long-term by reducing shoot growth, with complicated feedback processes mediating these distinct strategies (Tardieu et al., 2018). It may be that Si is only beneficial for a subset of these processes that vary between drought types. Overall, the contrasting yield responses to Si under different watering regimes warrants further attention so that farmers can make informed choices when applying Si fertilizers as a mitigation for drought stress.

## 5 Conclusions and future perspectives

We found that the impact of Si treatment on wheat water use and drought resilience is strongly affected by both genotype and watering regime, helping to explain why different studies have come to different conclusions on the potential benefits of Si. Our results further show that whilst stomatal density may play a role in the impact of Si treatment on g_s_ and stomatal function, stomatal density alone cannot be responsible for genotype-specific yield responses to Si. It is important for future work to characterize the mechanisms responsible for these genotype-specific or stress-specific responses. If Si-fertilization is to be used as an effective and robust agronomic practice, farmers need to know: (a) if their selected cultivar is one that can benefit from Si addition under drought stress; and (b) when and how to apply Si fertilizers to the field to achieve the best protection against different types of drought stress.

## Data Availability

The raw data supporting the conclusions of this article will be made available by the authors, without undue reservation.

## References

[B1] Agarie S. Uchida H. Agata W. Kubota F. Kaufman P. B. (1998). Effects of silicon on transpiration and leaf conductance in rice plants (*Oryza sativa* L.). Plant Production Sci. 1, 89–95. doi: 10.1626/pps.1.89

[B2] Ashfaq W. Brodie G. Fuentes S. Pang A. Gupta D. (2024). Silicon improves root system and canopy physiology in wheat under drought stress. Plant Soil 502, 279–296. doi: 10.1007/s11104-023-06202-4

[B3] Ayed S. Othmani A. Bouhaouel I. Rasâa N. Othmani S. Amara H. S. (2022). Effect of Silicon (Si) Seed Priming on Germination and Effectiveness of its Foliar Supplies on Durum Wheat (*Triticum turgidum* L. ssp. *durum*) Genotypes under Semi-Arid Environment. Silicon 14, 1731–1741. doi: 10.1007/s12633-021-00963-2

[B4] Barratt L. J. He Z. Fellgett A. Wang L. Mason S. M. Bancroft I.. (2023). Co-expression network analysis of diverse wheat landraces reveals markers of early thermotolerance and a candidate master regulator of thermotolerance genes. Plant J. 115, 614–626. doi: 10.1111/tpj.16248, PMID: 37077043 PMC10953029

[B5] Bukhari M. A. Ahmad Z. Ashraf M. Y. Afzal M. Nawaz F. Nafees M.. (2021). Silicon mitigates drought stress in wheat (*Triticum aestivum* L.) through improving photosynthetic pigments, biochemical and yield characters. Silicon 13, 4757–4772. doi: 10.1007/s12633-020-00797-4

[B6] Camargo M. S. Fernández Honaine M. Osterrieth M. Bozza N. G. da Mota Silva V. Benvenuto M. L.. (2021). Silicon fertilization increases gas-exchange and biomass by silicophytolith deposition in the leaves of contrasting drought-tolerant sugarcane cultivars under well-watered conditions. Plant Soil 466, 581–595. doi: 10.1007/s11104-021-05063-z

[B7] Challinor A. J. Watson J. Lobell D. B. Howden S. M. Smith D. R. Chhetri N. (2014). A meta-analysis of crop yield under climate change and adaptation. Nat. Climate Change 4, 287–291. doi: 10.1038/nclimate2153

[B8] Chen W. Yao X. Cai K. Chen J. (2011). Silicon alleviates drought stress of rice plants by improving plant water status, photosynthesis and mineral nutrient absorption. Biol. Trace Element Res. 142, 67–76. doi: 10.1007/s12011-010-8742-x, PMID: 20532668

[B9] Cheraghi M. Motesharezadeh B. Mousavi S. M. Basirat M. Alikhani H. A. Zarebanadkouki M. (2024). Application of silicon improves rhizosheath formation, morpho-physiological and biochemical responses of wheat under drought stress. Plant Soil 503, 263–281. doi: 10.1007/s11104-024-06584-z

[B10] Christian M. M. Shimelis H. Laing M. D. Tsilo T. J. (2023). The effect of silicon fertilizers on agronomic performance of bread wheat under drought stress and non-stress conditions. J. Agron. Crop Sci. 209, 827–840. doi: 10.1111/jac.12668

[B11] Christian M. M. Shimelis H. Laing M. D. Tsilo T. J. (2025). Association of yield and yield components among selected bread wheat genotypes under silicon fertilisation and drought conditions. J. Agron. Crop Sci. 211, e70020. doi: 10.1111/jac.70020

[B12] Cole M. B. Augustin M. A. Robertson M. J. Manners J. M. (2018). The science of food security. NPJ Sci. Food 2, 14. doi: 10.1038/s41538-018-0021-9, PMID: 31304264 PMC6550266

[B13] Cooke J. Carey J. C. (2023). Stress alters the role of silicon in controlling plant water movement. Funct. Ecol. 37, 2985–2999. doi: 10.1111/1365-2435.14447

[B14] Cooke J. Leishman M. R. (2016). Consistent alleviation of abiotic stress with silicon addition: a meta-analysis. Funct. Ecol. 30, 1340–1357. doi: 10.1111/1365-2435.12713

[B15] Coskun D. Britto D. T. Huynh W. Q. Kronzucker H. J. (2016). The role of silicon in higher plants under salinity and drought stress. Front. Plant Sci. 7. doi: 10.3389/fpls.2016.01072, PMID: 27486474 PMC4947951

[B16] Coskun D. Deshmukh R. Sonah H. Menzies J. G. Reynolds O. Ma J. F.. (2019). The controversies of silicon's role in plant biology. New Phytol. 221, 67–85. doi: 10.1111/nph.15343, PMID: 30007071

[B17] Debona D. Rodrigues F. A. Datnoff L. E. (2017). Silicon's role in abiotic and biotic plant stresses. Annu. Rev. Phytopathol. 55, 85–107. doi: 10.1146/annurev-phyto-080516-035312, PMID: 28504920

[B18] de Tombeur F. Raven J. A. Toussaint A. Lambers H. Cooke J. Hartley S. E.. (2023). Why do plants silicify? Trends Ecol. Evol. 38, 275–288. doi: 10.1016/j.tree.2022.11.002, PMID: 36428125

[B19] Dunn J. Hunt L. Afsharinafar M. Meselmani M. A. Mitchell A. Howells R.. (2019). Reduced stomatal density in bread wheat leads to increased water-use efficiency. J. Exp. Bot. 70, 4737–4748. doi: 10.1093/jxb/erz248, PMID: 31172183 PMC6760291

[B20] Epstein E. (1994). The anomaly of silicon in plant biology. Proc. Natl. Acad. Sci. 91, 11–17. doi: 10.1073/pnas.91.1.11, PMID: 11607449 PMC42876

[B21] Erenstein O. Jaleta M. Mottaleb K. A. Sonder K. Donovan J. Braun H.-J. (2022). Global Trends in Wheat Production, Consumption and Trade. in Reynolds M. P. Braun H. J. (eds) Wheat Improvement (Cham, Switzerland: Springer), 47–66. doi: 10.1007/978-3-030-90673-3_4

[B22] Farooq M. Hussain M. Siddique K. H. M. (2014). Drought stress in wheat during flowering and grain-filling periods. Crit. Rev. Plant Sci. 33, 331–349. doi: 10.1080/07352689.2014.875291

[B23] Foresti A. C. de Paula Quintão Scalon S. Santos C. C. Reis L. C. Linné J. A. (2024). Does silicon in *eugenia myrcianthes* seedlings under water stress contribute in the tolerance and recovery? J. Soil Sci. Plant Nutr. 24, 2208–2220. doi: 10.1007/s42729-024-01664-6

[B24] Fox J. Weisberg S. (2019). An R Companion to Applied Regression (Thousand Oaks CA: Sage). Available online at: https://www.john-fox.ca/Companion/.

[B25] Frew A. Weston L. A. Reynolds O. L. Gurr G. M. (2018). The role of silicon in plant biology: a paradigm shift in research approach. Ann. Bot. 121, 1265–1273. doi: 10.1093/aob/mcy009, PMID: 29438453 PMC6007437

[B26] Gao X. Zou C. Wang L. Zhang F. (2005). Silicon improves water use efficiency in maize plants. J. Plant Nutr. 27, 1457–1470. doi: 10.1081/PLN-200025865

[B27] Gao X. Zou C. Wang L. Zhang F. (2006). Silicon decreases transpiration rate and conductance from stomata of maize plants. J. Plant Nutr. 29, 1637–1647. doi: 10.1080/01904160600851494

[B28] Gocke M. Liang W. Sommer M. Kuzyakov Y. (2013). Silicon uptake by wheat: Effects of Si pools and pH. J. Plant Nutr. Soil Sci. 176, 551–560. doi: 10.1002/jpln.201200098

[B29] Guntzer F. Keller C. Meunier J.-D. (2012). Benefits of plant silicon for crops: a review. Agron. Sustain. Dev. 32, 201–213. doi: 10.1007/s13593-011-0039-8

[B30] Hattori T. Inanaga S. Araki H. An P. Morita S. Luxová M.. (2005). Application of silicon enhanced drought tolerance in *Sorghum bicolor* . Physiologia Plantarum 123, 459–466. doi: 10.1111/j.1399-3054.2005.00481.x

[B31] Hodson M. J. White P. J. Mead A. Broadley M. R. (2005). Phylogenetic variation in the silicon composition of plants. Ann. Bot. 96, 1027–1046. doi: 10.1093/aob/mci255, PMID: 16176944 PMC4247092

[B32] Huang S. Yamaji N. Sakurai G. Mitani-Ueno N. Konishi N. Ma J. F. (2022). A pericycle-localized silicon transporter for efficient xylem loading in rice. New Phytol. 234, 197–208. doi: 10.1111/nph.17959, PMID: 35020209

[B33] IPCC (2023). Climate change 2023: synthesis report. Contribution of working groups I, II and III to the sixth assessment report of the intergovernmental panel on climate change 35–115. doi: 10.59327/IPCC/AR6-9789291691647

[B34] Johnson S. N. Chen Z.-H. Rowe R. C. Tissue D. T. (2022). Field application of silicon alleviates drought stress and improves water use efficiency in wheat. Front. Plant Sci. 13. doi: 10.3389/fpls.2022.1030620, PMID: 36438110 PMC9682199

[B35] Johnson S. N. Waterman J. M. Hartley S. E. Cooke J. Ryalls J. M. W. Lagisz M.. (2024). Plant silicon defences suppress herbivore performance, but mode of feeding is key. Ecol. Lett. 27, e14519. doi: 10.1111/ele.14519, PMID: 39400424

[B36] Kim S. G. Kim K. W. Park E. W. Choi D. (2002). Silicon-induced cell wall fortification of rice leaves: A possible cellular mechanism of enhanced host resistance to blast. Phytopathology 92, 1095–1103. doi: 10.1094/phyto.2002.92.10.1095, PMID: 18944220

[B37] Kumar S. Milstein Y. Brami Y. Elbaum M. Elbaum R. (2017a). Mechanism of silica deposition in sorghum silica cells. New Phytol. 213, 791–798. doi: 10.1111/nph.14173, PMID: 27621091

[B38] Kumar S. Soukup M. Elbaum R. (2017b). Silicification in grasses: variation between different cell types. Front. Plant Sci. 8. doi: 10.3389/fpls.2017.00438, PMID: 28400787 PMC5368260

[B39] Lavinsky A. O. Detmann K. C. Reis J. V. Ávila R. T. Sanglard M. L. Pereira L. F.. (2016). Silicon improves rice grain yield and photosynthesis specifically when supplied during the reproductive growth stage. J. Plant Physiol. 206, 125–132. doi: 10.1016/j.jplph.2016.09.010, PMID: 27744227

[B40] Law C. Exley C. (2011). New insight into silica deposition in horsetail (*Equisetum arvense*). BMC Plant Biol. 11, 112. doi: 10.1186/1471-2229-11-112, PMID: 21801378 PMC3160890

[B41] Lenth R. V. (2024). emmeans: Estimated Marginal Means, aka Least-Squares Means (R package version 1.10.4). Available online at: https://CRAN.R-project.org/package=emmeans.

[B42] Lesk C. Rowhani P. Ramankutty N. (2016). Influence of extreme weather disasters on global crop production. Nature 529, 84–87. doi: 10.1038/nature16467, PMID: 26738594

[B43] Liang Y. Hua H. Zhu Y.-G. Zhang J. Cheng C. Römheld V. (2006). Importance of plant species and external silicon concentration to active silicon uptake and transport. New Phytol. 172, 63–72. doi: 10.1111/j.1469-8137.2006.01797.x, PMID: 16945089

[B44] Liang Y. Nikolic M. Bélanger R. Gong H. Song A. (2015). “Analysis of silicon in Soil, Plant and Fertilizer,” in Silicon in Agriculture: From Theory to Practice (Springer Netherlands, Dordrecht), 19–44.

[B45] Liu P. Yin L. Wang S. Zhang M. Deng X. Zhang S.. (2015). Enhanced root hydraulic conductance by aquaporin regulation accounts for silicon alleviated salt-induced osmotic stress in *Sorghum bicolor* L. Environ. Exp. Bot. 111, 42–51. doi: 10.1016/j.envexpbot.2014.10.006

[B46] Luyckx M. Hausman J.-F. Lutts S. Guerriero G. (2017). Silicon and plants: current knowledge and technological perspectives. Front. Plant Sci. 8. doi: 10.3389/fpls.2017.00411, PMID: 28386269 PMC5362598

[B47] Ma J. Kazuo N. Takahashi E. (1989). Effect of silicon on the growth of rice plant at different growth stages. Soil Sci. Plant Nutr. 35, 347–356. doi: 10.1080/00380768.1989.10434768

[B48] Ma J. F. Tamai K. Yamaji N. Mitani N. Konishi S. Katsuhara M.. (2006). A silicon transporter in rice. Nature 440, 688–691. doi: 10.1038/nature04590, PMID: 16572174

[B49] Ma J. F. Yamaji N. (2006). Silicon uptake and accumulation in higher plants. Trends Plant Sci. 11, 392–397. doi: 10.1016/j.tplants.2006.06.007, PMID: 16839801

[B50] Ma J. F. Yamaji N. (2015). A cooperative system of silicon transport in plants. Trends Plant Sci. 20, 435–442. doi: 10.1016/j.tplants.2015.04.007, PMID: 25983205

[B51] Ma J. F. Yamaji N. Mitani N. Tamai K. Konishi S. Fujiwara T.. (2007). An efflux transporter of silicon in rice. Nature 448, 209–212. doi: 10.1038/nature05964, PMID: 17625566

[B52] Malhi G. S. Kaur M. Kaushik P. (2021). Impact of climate change on agriculture and its mitigation strategies: A review. Sustainability 13, 1318. doi: 10.3390/su13031318

[B53] Malik M. A. Hassan S. Rashid I. Tahir I. (2025). Wheat genotypes vary in efficiently using silicon to enhance growth and yield– a physiological perspective. J. Soil Sci. Plant Nutr. 25, 3498–3507. doi: 10.1007/s42729-025-02348-5

[B54] Mandlik R. Thakral V. Raturi G. Shinde S. Nikolić M. Tripathi D. K.. (2020). Significance of silicon uptake, transport, and deposition in plants. J. Exp. Bot. 71, 6703–6718. doi: 10.1093/jxb/eraa301, PMID: 32592476

[B55] McLarnon E. McQueen-Mason S. Lenk I. Hartley S. E. (2017). Evidence for active uptake and deposition of si-based defenses in tall fescue. Front. Plant Sci. 8. doi: 10.3389/fpls.2017.01199, PMID: 28769939 PMC5513917

[B56] Mitani-Ueno N. Ma J. F. (2021). Linking transport system of silicon with its accumulation in different plant species. Soil Sci. Plant Nutr. 67, 10–17. doi: 10.1080/00380768.2020.1845972

[B57] Mitani-Ueno N. Yamaji N. Huang S. Yoshioka Y. Miyaji T. Ma J. F. (2023). A silicon transporter gene required for healthy growth of rice on land. Nat. Commun. 14, 6522. doi: 10.1038/s41467-023-42180-y, PMID: 37857615 PMC10587147

[B58] Motomura H. Fujii T. Suzuki M. (2004). Silica deposition in relation to ageing of leaf tissues in *sasa veitchii* (Carrière) rehder (Poaceae: bambusoideae). Ann. Bot. 93, 235–248. doi: 10.1093/aob/mch034, PMID: 14744706 PMC4242195

[B59] Pierantoni M. Tenne R. Brumfeld V. Kiss V. Oron D. Addadi L.. (2017). Plants and light manipulation: the integrated mineral system in okra leaves. Advanced Sci. 4, 1600416. doi: 10.1002/advs.201600416, PMID: 28546910 PMC5441490

[B60] Raven J. A. (1983). The transport and function of silicon in plants. Biol. Rev. 58, 179–207. doi: 10.1111/j.1469-185X.1983.tb00385.x

[B61] Ray D. K. Mueller N. D. West P. C. Foley J. A. (2013). Yield trends are insufficient to double global crop production by 2050. PloS One 8, e66428. doi: 10.1371/journal.pone.0066428, PMID: 23840465 PMC3686737

[B62] R Core Team (2024). R: A Language and Environment for Statistical Computing (Vienna, Austria: R Foundation for Statistical Computing). Available online at: https://www.R-project.org/.

[B63] Reidinger S. Ramsey M. H. Hartley S. E. (2012). Rapid and accurate analyses of silicon and phosphorus in plants using a portable X-ray fluorescence spectrometer. New Phytol. 195, 699–706. doi: 10.1111/j.1469-8137.2012.04179.x, PMID: 22671981

[B64] Sakai W. S. Thom M. (1979). Localization of silicon in specific cell wall layers of the stomatal apparatus of sugar cane by use of energy dispersive X-ray analysis. Ann. Bot. 44, 245–248. doi: 10.1093/oxfordjournals.aob.a085725

[B65] Sauer D. Saccone L. Conley D. J. Herrmann L. Sommer M. (2006). Review of methodologies for extracting plant-available and amorphous Si from soils and aquatic sediments. Biogeochemistry 80, 89–108. doi: 10.1007/s10533-005-5879-3

[B66] Schneider C. A. Rasband W. S. Eliceiri K. W. (2012). NIH Image to ImageJ: 25 years of image analysis. Nat. Methods 9, 671–675. doi: 10.1038/nmeth.2089, PMID: 22930834 PMC5554542

[B67] Shamshiripour M. Motesharezadeh B. Rahmani H. A. Alikhani H. A. Etesami H. (2022). Optimal concentrations of silicon enhance the growth of soybean (*Glycine max* L.) cultivars by improving nodulation, root system architecture, and soil biological properties. Silicon 14, 5333–5345. doi: 10.1007/s12633-021-01273-3

[B68] Tamai K. Ma J. F. (2008). Reexamination of silicon effects on rice growth and production under field conditions using a low silicon mutant. Plant Soil 307, 21–27. doi: 10.1007/s11104-008-9571-y

[B69] Tardieu F. Simonneau T. Muller B. (2018). The physiological basis of drought tolerance in crop plants: A scenario-dependent probabilistic approach. Annu. Rev. Plant Biol. 69, 733–759. doi: 10.1146/annurev-arplant-042817-040218, PMID: 29553801

[B70] Thakral V. Raturi G. Sudhakaran S. Mandlik R. Sharma Y. Shivaraj S. M.. (2024). Silicon, a quasi-essential element: Availability in soil, fertilizer regime, optimum dosage, and uptake in plants. Plant Physiol. Biochem. 208, 108459. doi: 10.1016/j.plaphy.2024.108459, PMID: 38484684

[B71] Thorne S. J. Hartley S. E. Maathuis F. J. M. (2020). Is silicon a panacea for alleviating drought and salt stress in crops? Front. Plant Sci. 11. doi: 10.3389/fpls.2020.01221, PMID: 32973824 PMC7461962

[B72] Thorne S. J. Hartley S. E. Maathuis F. J. M. (2021). The effect of silicon on osmotic and drought stress tolerance in wheat landraces. Plants 10, 814. doi: 10.3390/plants10040814, PMID: 33924159 PMC8074377

[B73] Thorne S. J. Maathuis F. J. M. Hartley S. E. (2023). Induction of silicon defences in wheat landraces is local, not systemic, and driven by mobilization of soluble silicon to damaged leaves. J. Exp. Bot. 74, 5363–5373. doi: 10.1093/jxb/erad224, PMID: 37314063

[B74] Ueno O. Agarie S. (2005). Silica deposition in cell walls of the stomatal apparatus of rice leaves. Plant Production Sci. 8, 71–73. doi: 10.1626/pps.8.71

[B75] Vandegeer R. K. Zhao C. Cibils-Stewart X. Wuhrer R. Hall C. R. Hartley S. E.. (2021). Silicon deposition on guard cells increases stomatal sensitivity as mediated by K+ efflux and consequently reduces stomatal conductance. Physiologia Plantarum 171, 358–370. doi: 10.1111/ppl.13202, PMID: 32880970

[B76] Wickham H. (2016). ggplot2: Elegant Graphics for Data Analysis (New York: Springer-Verlag).

[B77] Yamaji N. Ma J. F. (2009). A transporter at the node responsible for intervascular transfer of silicon in rice. Plant Cell 21, 2878–2883. doi: 10.1105/tpc.109.069831, PMID: 19734433 PMC2768918

[B78] Yamaji N. Mitatni N. Ma J. F. (2008). A transporter regulating silicon distribution in rice shoots. Plant Cell 20, 1381–1389. doi: 10.1105/tpc.108.059311, PMID: 18515498 PMC2438455

[B79] Yamaji N. Sakurai G. Mitani-Ueno N. Ma J. F. (2015). Orchestration of three transporters and distinct vascular structures in node for intervascular transfer of silicon in rice. Proc. Natl. Acad. Sci. 112, 11401–11406. doi: 10.1073/pnas.1508987112, PMID: 26283388 PMC4568664

[B80] Zampieri M. Ceglar A. Dentener F. Toreti A. (2017). Wheat yield loss attributable to heat waves, drought and water excess at the global, national and subnational scales. Environ. Res. Lett. 12, 064008. doi: 10.1088/1748-9326/aa723b

[B81] Zhang J. Zhang S. Cheng M. Jiang H. Zhang X. Peng C.. (2018). Effect of drought on agronomic traits of rice and wheat: A meta-analysis. Int. J. Environ. Res. Public Health 15, 839. doi: 10.3390/ijerph15050839, PMID: 29695095 PMC5981878

