# Supplementary material for: The beneficial impact of silicon on wheat drought resilience is dependent on cultivar and stress intensity
Source: Front Plant Sci. 2025 Aug 21;16:1661405. doi: 10.3389/fpls.2025.1661405 (PMC12408560; doi:10.3389/fpls.2025.1661405)
Supplement: Supplementary file 1 [file DataSheet1.zip › Supplementary Figures.docx]

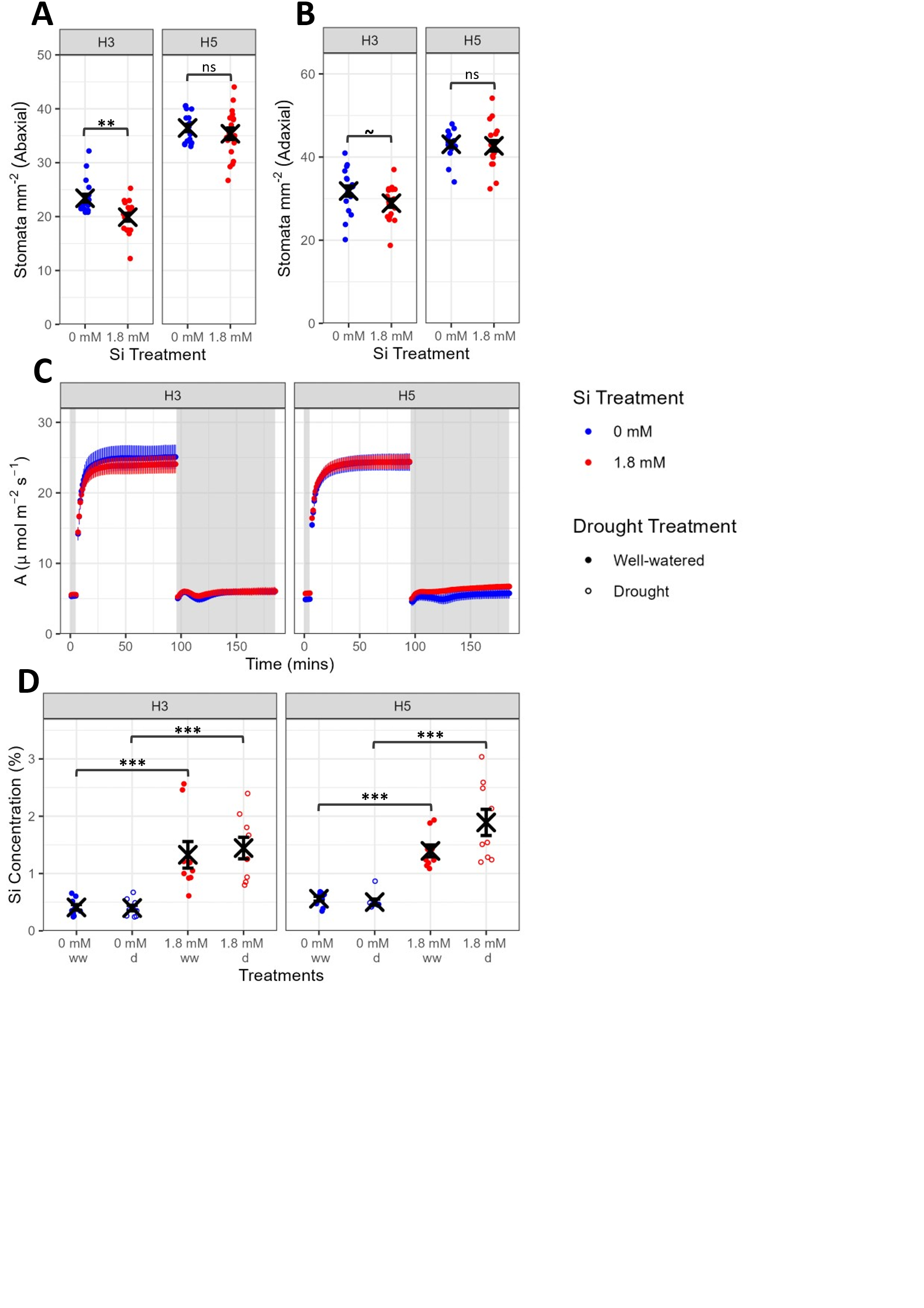
Supplementary Figures

**Supplementary Figure 1.** Impact of Si treatment and drought treatment on high Si accumulating genotypes H3 and H5 (Exp1). **(A)** Abaxial and **(B)** Adaxial stomatal density of leaf 6 on 6-week-old plants (n=17-18). **(C)** Response of A to shifts in light intensity, measured by infra-red gas analysis of leaf 6 on 6-week-old plants (n=5-6). Grey-shaded background represents 100 PAR (low light) and white background 1000 PAR (high light). **(D)** Si concentration measured on leaf samples collected post-drought during week 11 (n=8-9). Blue points represent 0 mM Si treatment, red points represent 1.8 mM Si treatment. Closed points represent well-watered plants, open points represent droughted plants. Mean values ± SE are shown. The emmeans() package in R was used to test for statistically significant pairwise differences in parameter means between the 0 mM and 1.8 mM Si treatments for each genotype and drought treatment. ns non-significant, P<0.1 ~, P<0.01 **, P<0.001 ***.


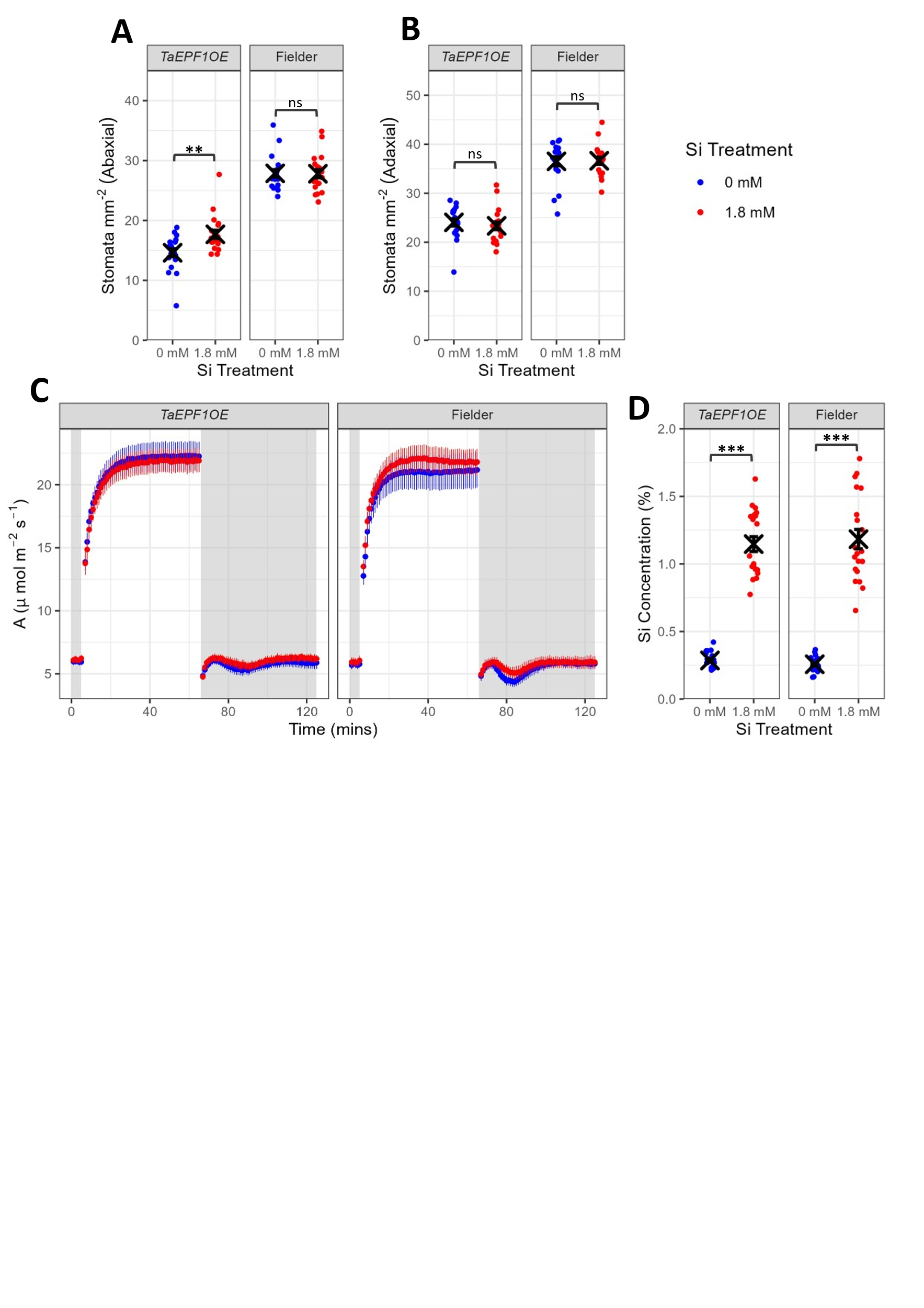
**Supplementary Figure 2.** Impact of Si on reduced stomatal density mutant (*TaEPF1OE*) and non-transgenic Fielder control genotype (Exp2). **(A)** Abaxial and **(B)** Adaxial stomatal density of leaf 5 on 5-6-week-old plants (n=20). **(C)** Response of A to shifts in light intensity, measured using infra-red gas analysis on leaf 5 during weeks 5-6 (n=6). Grey-shaded background represents 100 PAR (low light) and white background 1000 PAR (high light). **(D)** Si concentration measured on leaf samples collected pre-drought during week 7 (n=20). Blue points represent 0 mM Si treatment, red points represent 1.8 mM Si treatment. Mean values ± SE are shown. The emmeans() package in R was used to test for statistically significant pairwise differences in parameter means between the 0 mM and 1.8 mM Si treatments for each genotype. ns non-significant, P<0.01 **, P<0.001 ***.


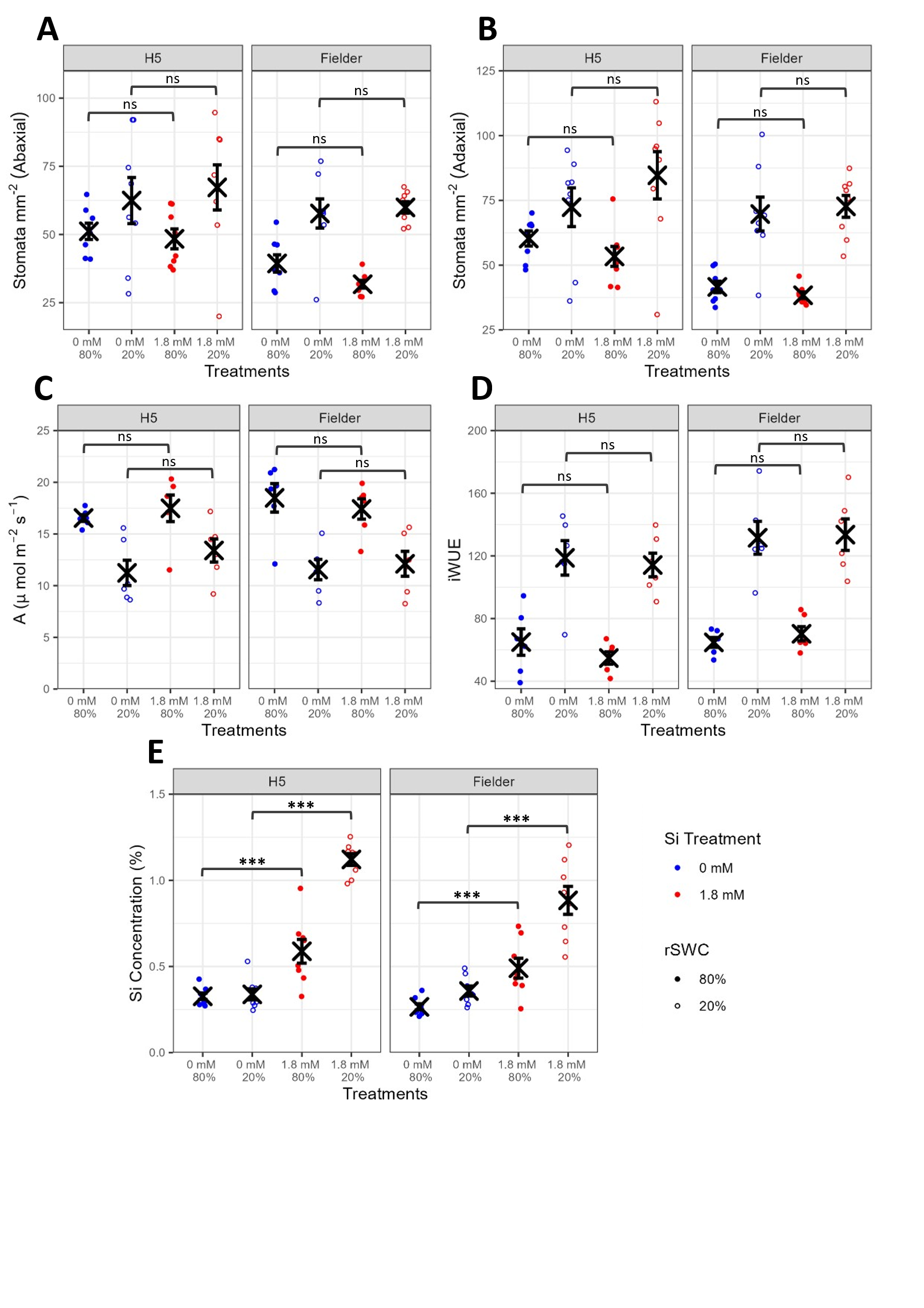
**Supplementary Figure 3.** Impact of Si treatment and contrasting rSWC on H5 and Fielder genotypes (Exp3). **(A)** Abaxial and **(B)** Adaxial stomatal density of leaf 6 for H5, leaf 5 for Fielder on 6-week-old plants (n=8). Steady-state **(C)** A and **(D)** iWUE, measured using infra-red gas analysis on leaf 6 for H5, leaf 5 for Fielder during week 6 (n=6). **(E)** Si concentration measured on leaf samples collected during week 6 (n=8). Blue points represent 0 mM Si treatment, red points represent 1.8 mM Si treatment. Closed points represent 80% rSWC treatment, open points represent 20% rSWC treatment. Mean values ± SE are shown. The emmeans() package in R was used to test for statistically significant pairwise differences in parameter means between the 0 mM and 1.8 mM Si treatments for each genotype and drought treatment. ns non-significant, P<0.001 ***.
