# Supplementary material for: The beneficial impact of silicon on wheat drought resilience is dependent on cultivar and stress intensity
Source: Front Plant Sci. 2025 Aug 21;16:1661405. doi: 10.3389/fpls.2025.1661405 (PMC12408560; doi:10.3389/fpls.2025.1661405)
Supplement: Supplementary file 1 [file DataSheet1.zip › Supplementary Tables.docx]

Supplementary Tables

Note that for all supplementary tables, F and t values have been rounded to 1 decimal place, P values have been rounded to 3 significant figures or <0.0001 where appropriate (P<0.1 ~, P<0.05 *, P<0.01 **, P<0.001 ***). If data were transformed to meet ANOVA assumptions, or did not satisfy ANOVA assumptions, this is noted in the relevant supplementary table.

**Supplementary Table 1: Exp1, two-way ANOVAs.** Two-way ANOVA results testing for the impact of genotype and Si treatment on various parameters measured during Exp1.

| Parameter | df | Genotype (G) | | Si Treatment (Si) | | G x Si | |
| --- | --- | --- | --- | --- | --- | --- | --- |
|  |  | F value | P value | F value | P value | F value | P value |
| Total stomatal density | 1,67 | 256.0 | <0.0001  *** | 5.7 | 0.0201  * | 2.2 | 0.141 |
| Abaxial stomatal density | 1,67 | 296.4 | <0.0001  *** | 7.8 | 0.00677  ** | 2.2 | 0.145 |
| Adaxial stomatal density | 1,67 | 128.9 | <0.0001  *** | 2.3 | 0.134 | 1.3 | 0.252 |
| Initial g_min_ | 1,18 | 37.7 | <0.0001  *** | 6.1 | 0.0233  * | 0.0 | 0.864 |
| Closing g_min_ *(log-transformed but still non-normal)* | 1,18 | 20.3 | 0.000275  *** | 10.2 | 0.00498  ** | 2.9 | 0.108 |
| Total water use during drought | 1,31 | 0.3 | 0.587 | 27.0 | <0.0001  *** | 0.2 | 0.691 |

**Supplementary Table 2: Exp1, three-way ANOVAs.** Three-way ANOVA results testing for the impact of genotype, Si treatment and drought treatment on various parameters measured during Exp1.

| Parameter | df | Genotype (G) | | Si Treatment (Si) | | Drought Treatment (D) | | G x Si | | G x D | | Si x D | | G x Si x D | |
| --- | --- | --- | --- | --- | --- | --- | --- | --- | --- | --- | --- | --- | --- | --- | --- |
|  |  | F value | P value | F value | P value | F value | P value | F value | P value | F value | P value | F value | P value | F value | P value |
| Silicon concentration *(log-transformed)* | 1,63 | 11.4 | 0.00126  ** | 204.1 | <0.0001  *** | 0.6 | 0.432 | 0.4 | 0.535 | 0.1 | 0.812 | 2.6 | 0.109 | 0.5 | 0.479 |
| Aboveground biomass | 1,62 | 4.8 | 0.0322  * | 1.1 | 0.295 | 499.2 | <0.0001  *** | 6.8 | 0.0113  * | 12.7 | 0.000708  *** | 1.6 | 0.209 | 1.4 | 0.234 |
| Total seed mass | 1,62 | 0.3 | 0.602 | 0.2 | 0.633 | 1041.7 | <0.0001  *** | 6.5 | 0.0131  * | 0.9 | 0.335 | 1.2 | 0.269 | 3.6 | 0.0634  ~ |

**Supplementary Table 3: Exp1, *post-hoc* testing, 0 mM Si vs 1.8 mM Si.** *Post-hoc* emmeans() results, testing for statistically significant pairwise differences in parameter means between the 0 mM and 1.8 mM Si treatments for each genotype in Exp1.

| Parameter | df | H3 | | H5 | |
| --- | --- | --- | --- | --- | --- |
|  |  | T ratio | P value | T ratio | P value |
| Total stomatal density | 67 | 2.7 | 0.0078  ** | 0.6 | 0.544 |
| Abaxial stomatal density | 67 | 3.0 | 0.0035  ** | 0.9 | 0.366 |
| Adaxial stomatal density | 67 | 1.9 | 0.0631  ~ | 0.2 | 0.810 |
| Initial g_min_ | 18 | -1.6 | 0.121 | -1.9 | 0.0771  ~ |
| Closing g_min_ *(log-transformed but still non-normal)* | 18 | -1.1 | 0.301 | -3.5 | 0.0028  ** |
| Total water use during drought | 31 | 3.4 | 0.0016  ** | 3.9 | 0.0005  *** |

**Supplementary Table 4: Exp1, *post-hoc* testing, 0 mM Si vs 1.8 mM Si.** *Post-hoc* emmeans() results, testing for statistically significant pairwise differences in parameter means between the 0 mM and 1.8 mM Si treatments for each genotype/drought treatment in Exp1.

| Parameter | df | H3 well-watered | | H3 drought | | H5 well-watered | | H5 drought | |
| --- | --- | --- | --- | --- | --- | --- | --- | --- | --- |
|  |  | T ratio | P value | T ratio | P value | T ratio | P value | T ratio | P value |
| Si concentration *(log-transformed)* | 63 | -7.0 | <0.0001  *** | -8.0 | <0.0001  *** | -5.7 | <0.0001 *** | -7.8 | <0.0001  *** |
| Aboveground biomass | 62 | -0.7 | 0.482 | 1.7 | 0.0855  ~ | -2.3 | 0.0260  * | -2.1 | 0.0357  * |
| Total seed mass | 62 | 0.2 | 0.839 | 1.0 | 0.340 | -0.6 | 0.580 | -3.5 | 0.0009  *** |

**Supplementary Table 5: Exp2, two-way ANOVAs.** Two-way ANOVA results testing for the impact of genotype and Si treatment on various parameters measured during Exp2.

| Parameter | df | Genotype (G) | | Si Treatment (Si) | | G x Si | |
| --- | --- | --- | --- | --- | --- | --- | --- |
|  |  | F value | P value | F value | P value | F value | P value |
| Total stomatal density *(log-transformed but still non-normal)* | 1,76 | 472.2 | <0.0001  *** | 1.7 | 0.196 | 1.8 | 0.188 |
| Abaxial stomatal density *(non-normal)* | 1,76 | 314.9 | <0.0001  *** | 5.2 | 0.0249  * | 5.7 | 0.0195  * |
| Adaxial stomatal density  *(non-normal)* | 1,76 | 271.2 | <0.0001  *** | 0.1 | 0.705 | 0.3 | 0.602 |
| Initial g_min_ | 1,20 | 2.5 | 0.132 | 1.5 | 0.236 | 0.8 | 0.379 |
| Closing g_min_ | 1,20 | 7.1 | 0.0150  * | 3.2 | 0.0896  ~ | 0.7 | 0.403 |
| Si concentration *(log-transformed)* | 1,76 | 0.8 | 0.377 | 824.8 | <0.0001  *** | 1.5 | 0.218 |
| Total water use during drought | 1,36 | 0.0 | 0.837 | 1.3 | 0.259 | 0.0 | 0.860 |

**Supplementary Table 6: Exp2, three-way ANOVAs.** Three-way ANOVA results testing for the impact of genotype, Si treatment and drought treatment on various parameters measured during Exp2.

| Parameter | df | Genotype (G) | | Si Treatment (Si) | | Drought Treatment (D) | | G x Si | | G x D | | Si x D | | G x Si x D | | |
| --- | --- | --- | --- | --- | --- | --- | --- | --- | --- | --- | --- | --- | --- | --- | --- | --- |
|  |  | F value | P value | F value | P value | F value | P value | F value | P value | F value | P value | F value | P value | | F value | P value |
| Aboveground biomass *(log-transformed)* | 1,72 | 38.3 | <0.0001  *** | 29.0 | <0.0001  *** | 769.0 | <0.0001  *** | 3.8 | 0.0551  ~ | 1.8 | 0.178 | 1.3 | 0.256 | | 0.1 | 0.709 |
| Total seed mass | 1,72 | 71.3 | <0.0001  *** | 28.8 | <0.0001  *** | 794.4 | <0.0001  *** | 1.2 | 0.279 | 6.1 | 0.0155  * | 0.0 | 0.975 | | 0.0 | 0.997 |

**Supplementary Table 7: Exp2, *post-hoc* testing, 0 mM Si vs 1.8 mM Si.** *Post-hoc* emmeans() results, testing for statistically significant pairwise differences in parameter means between the 0 mM and 1.8 mM Si treatments for each genotype in Exp2.

| Parameter | df | *TaEPF1OE* | | Fielder | |
| --- | --- | --- | --- | --- | --- |
|  |  | T ratio | P value | T ratio | P value |
| Total stomatal density *(log-transformed but still non-normal)* | 76 | -1.9 | 0.0664  ~ | 0.0 | 0.987 |
| Abaxial stomatal density  *(non-normal)* | 76 | -3.3 | 0.0015  ** | 0.1 | 0.945 |
| Adaxial stomatal density  *(non-normal)* | 76 | 0.6 | 0.525 | -0.1 | 0.919 |
| Initial g_min_ | 20 | -0.2 | 0.822 | -1.5 | 0.149 |
| Closing g_min_ | 20 | -0.7 | 0.518 | -1.9 | 0.0769  ~ |
| Si concentration *(log-transformed)* | 76 | -19.4 | <0.0001  *** | -21.2 | <0.0001  *** |
| Total water use during drought | 36 | 0.7 | 0.498 | 0.9 | 0.355 |

**Supplementary Table 8: Exp2, *post-hoc* testing, 0 mM Si vs 1.8 mM Si.** *Post-hoc* emmeans() results, testing for statistically significant pairwise differences in parameter means between the 0 mM and 1.8 mM Si treatments for each genotype/drought treatment in Exp2.

| Parameter | df | *TaEPF1OE* well-watered | | *TaEPF1OE* drought | | Fielder well-watered | | Fielder drought | |
| --- | --- | --- | --- | --- | --- | --- | --- | --- | --- |
|  |  | T ratio | P value | T ratio | P value | T ratio | P value | T ratio | P value |
| Aboveground biomass *(log-transformed)* | 72 | -3.3 | 0.0016  ** | -4.1 | 0.0001  *** | -1.0 | 0.341 | -2.5 | 0.0155  * |
| Total seed mass | 72 | -3.2 | 0.0020  ** | -3.2 | 0.0018  ** | -2.1 | 0.0371  * | -2.2 | 0.0348  * |

**Supplementary Table 9: Exp3, three-way ANOVAs.** Three-way ANOVA results testing for the impact of genotype, Si treatment and rSWC on various parameters measured during Exp3.

| Parameter | df | Genotype (G) | | Si Treatment (Si) | | rSWC (D) | | G x Si | | G x D | | Si x D | | G x Si x D | |
| --- | --- | --- | --- | --- | --- | --- | --- | --- | --- | --- | --- | --- | --- | --- | --- |
|  |  | F value | P value | F value | P value | F value | P value | F value | P value | F value | P value | F value | P value | F value | P value |
| Total stomatal density *(non-normal)* | 1,56 | 10.0 | 0.00247  ** | 0.0 | 0.949 | 42.4 | <0.0001  *** | 0.2 | 0.642 | 1.6 | 0.211 | 2.3 | 0.132 | 0.1 | 0.702 |
| Abaxial stomatal density *(non-normal)* | 1,56 | 7.9 | 0.00673  ** | 0.1 | 0.817 | 28.4 | <0.0001  *** | 0.3 | 0.606 | 1.3 | 0.260 | 1.5 | 0.231 | 0.0 | 0.875 |
| Adaxial stomatal density *(non-normal)* | 1,56 | 10.2 | 0.00233  ** | 0.1 | 0.735 | 48.8 | <0.0001  *** | 0.1 | 0.711 | 1.6 | 0.213 | 2.8 | 0.0989  ~ | 0.7 | 0.392 |
| Steady-state g_s_ | 1,40 | 1.9 | 0.181 | 0.2 | 0.681 | 91.8 | <0.0001  *** | 1.6 | 0.207 | 0.1 | 0.706 | 0.0 | 0.949 | 1.0 | 0.331 |
| Steady-state A | 1,40 | 0.1 | 0.769 | 0.7 | 0.414 | 47.7 | <0.0001  *** | 1.3 | 0.254 | 0.8 | 0.368 | 0.8 | 0.367 | 0.0 | 0.898 |
| Steady-state iWUE | 1,40 | 4.4 | 0.0413  * | 0.1 | 0.748 | 116.8 | <0.0001  *** | 1.0 | 0.329 | 0.6 | 0.457 | 0.0 | 0.925 | 0.2 | 0.687 |
| Total water added *(log-transformed)* | 1,56 | 0.3 | 0.575 | 0.3 | 0.604 | 8993.6 | <0.0001  *** | 0.1 | 0.784 | 0.0 | 0.996 | 0.1 | 0.809 | 0.0 | 0.836 |
| Si concentration *(log-transformed)* | 1,56 | 6.1 | 0.0169  * | 176.5 | <0.0001  *** | 43.1 | <0.0001  *** | 1.5 | 0.225 | 0.6 | 0.437 | 16.5 | 0.000156  *** | 2.1 | 0.153 |
| Aboveground biomass *(log-transformed)* | 1,56 | 13.2 | 0.00062  *** | 4.9 | 0.0304  * | 1744.2 | <0.0001  *** | 0.6 | 0.456 | 0.9 | 0.346 | 0.4 | 0.509 | 0.1 | 0.815 |

**Supplementary Table 10: Exp3, *post-hoc* testing, 0 mM Si vs 1.8 mM Si.** *Post-hoc* emmeans() results, testing for statistically significant pairwise differences in parameter means between the 0 mM and 1.8 mM Si treatments for each genotype/rSWC in Exp3.

| Parameter | df | H5 80% | | H5 20% | | Fielder 80% | | Fielder 20% | |
| --- | --- | --- | --- | --- | --- | --- | --- | --- | --- |
|  |  | T ratio | P value | T ratio | P value | T ratio | P value | T ratio | P value |
| Total stomatal density *(non-normal)* | 56 | 0.7 | 0.492 | -1.2 | 0.226 | 0.8 | 0.443 | -0.4 | 0.712 |
| Abaxial stomatal density *(non-normal)* | 56 | 0.4 | 0.703 | -0.7 | 0.506 | 1.1 | 0.294 | -0.3 | 0.758 |
| Adaxial stomatal density *(non-normal)* | 56 | 0.9 | 0.365 | -1.6 | 0.109 | 0.4 | 0.673 | -0.4 | 0.697 |
| Steady-state g_s_ | 40 | -1.3 | 0.199 | -0.4 | 0.700 | 1.0 | 0.344 | -0.1 | 0.929 |
| Steady-state A | 40 | -0.6 | 0.551 | -1.4 | 0.174 | 0.7 | 0.496 | -0.4 | 0.725 |
| Steady-state iWUE | 40 | 0.9 | 0.370 | 0.4 | 0.688 | -0.5 | 0.629 | -0.2 | 0.861 |
| Total water added *(log-transformed)* | 56 | -0.1 | 0.919 | 0.3 | 0.729 | 0.4 | 0.704 | 0.4 | 0.679 |
| Si concentration *(log-transformed)* | 56 | -4.5 | <0.0001  *** | -10.0 | <0.0001  *** | -4.7 | <0.0001  *** | -7.3 | <0.0001  *** |
| Aboveground biomass *(log-transformed)* | 56 | 0.5 | 0.606 | 1.0 | 0.346 | 1.0 | 0.305 | 1.9 | 0.0579  ~ |
